# Lipidomic Remodelling and Low Sperm Quality Associated with High-Fat Diet-Induced Gut Microbial Dysbiosis in Mice

**DOI:** 10.3390/antiox15101281

**Published:** 2026-10-03

**Authors:** Melody Pan, Eleonora Scarlata, Fiona Hui, Jianguo Xia, Cristian O’Flaherty

**Affiliations:** 1 Department of Pharmacology and Therapeutics, McGill University, Montreal, QC H3G 1Y6, Canada; 2 Department of Surgery (Urology Division), McGill University, Montreal, QC H3G 1A4, Canada; 3 The Research Institute, McGill University Health Centre, Montreal, QC H3H 2R9, Canada; 4 Institute of Parasitology, McGill University, Montreal, QC H9X 3V9, Canada; 5 Department of Microbiology and Immunology, McGill University, Montreal, QC H3A 2B4, Canada; 6 Department of Anatomy and Cell Biology, McGill University, Montreal, QC H3A 0C7, Canada

**Keywords:** male infertility, gut microbiome dysbiosis, oxidative stress, lipidomics, gut–testis axis

## Abstract

Infertility affects one in six couples worldwide, with male infertility contributing to ~50% of cases. Rising obesity rates have been linked to impaired male fertility, potentially through alterations in the gut microbiota; however, underlying mechanisms remain poorly understood. We characterized gut microbiome composition and lipidomic profiles in stool, serum, and testis samples. We assessed sperm quality in C57BL6 male mice fed a 45% fat diet (high-fat diet, HFD) or a 10% fat diet (normal diet, ND). We analyzed the gut microbiota by 16S rRNA sequencing. We measured short-chain fatty acids by gas chromatography. We performed untargeted lipidomic profiling of stool, serum, and testis using UPLC-MS/MS. CASA and cytochemical and immunocytochemical analyses assessed sperm quality and oxidative stress markers. HFD-fed mice exhibited gut microbiota dysbiosis characterized by an increased Bacillota/Bacteroidota ratio, altered bacterial community composition, increased acetic acid, and reduced butyric acid. Lipidomic analyses revealed distinct stool, serum, and testis lipid profiles, including increased abundance of gut microbiota-associated lipids containing odd-chain fatty acyl groups in HFD-fed mice. These alterations were accompanied by reduced sperm motility, impaired acrosomal integrity, epididymal sperm maturation and sperm capacitation, and elevated oxidative and nitrosative stress. Collectively, these findings support a gut–testis–epididymis axis in which altered gut microbiota-derived lipids contribute to oxidative stress and reduced sperm quality.

## 1. Introduction

Infertility, defined as the inability to achieve pregnancy after one year of regular, unprotected sexual intercourse, affects approximately one in six couples worldwide [1]. Male-related factors contribute to nearly half of all infertility cases and arise from diverse genetic, hormonal, environmental, and lifestyle influences [2,3]. Standard evaluation of male infertility involves clinical history, physical examination, hormonal profiling, and semen analysis [4]. However, conventional semen analysis has limited predictive value because it does not routinely assess key functional parameters such as oxidative stress (OS) status. OS is recognized as a major contributor to male infertility, as excessive reactive oxygen species (ROS) can impair sperm membrane integrity, reduce motility, promote DNA damage, and compromise fertilization potential [5]. Oxidative stress disrupts sperm capacitation, an essential process characterized by a series of biochemical and molecular changes that allow the spermatozoon to acquire fertilizing ability [6,7]. Human spermatozoa with a high content of sphingolipids, like sphingosine and ceramide, display oxidative damage, promoting mitochondrial dysfunction, DNA oxidation and impaired sperm capacitation [8]. Notably, approximately 34% of male infertility cases remain idiopathic, highlighting the limitations of current diagnostic approaches and the presence of underlying biological mechanisms that remain poorly understood [9]. Among the emerging factors linked to male infertility, increasing attention has been directed toward the role of metabolic health, particularly the gut microbiome, in male reproductive function.

Obesity is a major global health concern, with prevalence rates rising globally in both adolescents and adults [10]. Beyond its well-established link to metabolic dysfunction and OS [11], obesity is associated with significant alterations in gut microbial composition, including reduced microbial diversity and shifts in community structure. This condition is called gut microbial dysbiosis [12,13]. The gut microbiome is a complex ecosystem composed of bacteria, archaea, fungi, and viruses that inhabit the gastrointestinal tract [14]. This microbial community plays an essential role in regulating host metabolism [15], immune function [16] and endocrine signalling [17].

The gut microbiota contributes to systemic lipid homeostasis by producing and transforming bioactive lipids and by modulating host lipid metabolism. In obesity, gut microbial dysbiosis may therefore alter both the production of microbiota-derived lipids and host lipid metabolic pathways, contributing to systemic lipid dysregulation [18,19]. Consequently, gut microbial dysbiosis can promote systemic inflammation, metabolic dysfunction, and OS [20,21], all of which have been implicated in male reproductive dysfunction [22,23]. In line with this, obese men commonly exhibit impaired sperm quality, including reduced sperm concentration, motility, and viability, as well as increased rates of oligozoospermia and asthenozoospermia [24,25].

Emerging studies have begun to investigate the role of gut microbial dysbiosis in male reproductive health, suggesting a gut–testis axis. In particular, evidence highlights the potential influence of gut microbiota-derived metabolites, such as short-chain fatty acids (SCFAs), on testicular development, spermatogenesis, and sperm function [26]. The reduction in sperm motility or asthenozoospermia has been associated with the loss of gut bacteria producing butyric acid [27]. Furthermore, studies transferring fecal microbiota from disease models associated with gut microbial dysbiosis into healthy recipients recapitulated key features of reproductive impairment, including reduced sperm motility and impaired spermatogenesis [28]. Conversely, restoring microbial balance has been associated with improvements in male fertility parameters [29]. However, a critical knowledge gap remains, as most studies are correlative and fail to establish direct mechanistic links between gut microbial dysbiosis and male infertility. Therefore, further investigation is required to elucidate the causal mechanisms underlying the gut–testis axis. Identifying the microbial metabolites and associated pathways that mediate male reproductive dysfunction is essential to understand how gut microbial dysbiosis contributes to impaired sperm function and to develop novel therapeutic targets.

Therefore, this study aims to investigate the mechanistic relationship between gut microbial dysbiosis and male reproductive dysfunction using a high-fat diet (HFD)-induced mouse model of gut dysbiosis. We found that HFD-induced gut microbiome dysbiosis promotes male infertility by producing abnormal metabolic products that induce oxidative stress. Specifically, we compared gut microbiome signatures between HFD- and normal diet (ND)-fed male mice, evaluated HFD effects on spermatogenesis, epididymal maturation, and sperm quality, and characterized gut microbiota-generated lipidomic products and their effects on reproductive function.

## 2. Materials and Methods

### 2.1. Chemicals and Reagents

Mouse monoclonal anti-8-hydroxy-2′-deoxyguanosine (8-OHdG) conjugated with fluorescein isothiocyanate (FITC), rabbit polyclonal anti-peroxiredoxin 6-SO_3_ (PRDX 6-SO_3_), mouse monoclonal anti-inducible nitric oxide synthase (iNOS), mouse monoclonal anti-3-nitrotyrosine (3-NT), and rabbit polyclonal anti-superoxide dismutase 1 (SOD1) antibodies were purchased from Abcam (Toronto, ON, Canada). Goat polyclonal anti-4-hydroxynonenal (4-HNE) antibody was purchased from Sigma-Aldrich (St. Louis, MO, USA). Goat polyclonal anti-mouse IgG (H + L) conjugated with Alexa Fluor 555, donkey polyclonal anti-goat IgG (H + L) conjugated with Alexa Fluor 555, and goat anti-rabbit IgG (H + L) conjugated with Alexa Fluor 555 antibodies were purchased from Invitrogen (Fisher Scientific, Ottawa, ON, Canada). Solutions of 10% formaldehyde and 100% xylene were purchased from ThermoFisher Scientific (Markham, ON, Canada). Gibco Giemsa stain was purchased from Fisher Scientific (Toronto, ON, Canada). Periodic acid solution, Schiff reagent, and hematoxylin were purchased from Sigma-Aldrich (St. Louis, MO, USA). Bluing solution was purchased from Surgipath (Concord, ON, Canada). High-performance liquid chromatography (HPLC)-grade water, methyl tert-butyl ether (MTBE), methanol, acetonitrile, formic acid, and toluene were purchased from Fisher Chemical (Morris Plains, NJ, USA). Testosterone and estradiol enzyme-linked immunosorbent assays were purchased from Elabscience (Houston, TX, USA).

### 2.2. Animals and Treatment

All experimental and animal handling protocols were approved by the Animal Care Committee at the Research Institute of the McGill University Health Centre (AUP # MUHC-5656), and the study adhered to regulations stipulated by the Canadian Council on Animal Care. C57BL/6 mice were born and maintained at the Animal Resources Division, Research Institute of McGill University Health Centre on a 14 h light/10 h dark cycle, with food and water provided with their dams until weaning at postnatal day 21. At that time, littermate male mice were randomly assigned to ND (*n* = 39) or HFD (*n* = 38) groups. Mice were fed an ND (D12450H, 10% fat, Research Diets, New Brunswick, NJ, USA) or an HFD (D12451, 45% fat, Research Diets, NJ, USA) until 8 weeks of age, covering the pre- and post-pubertal period through adulthood. We recorded body weight and food consumption weekly (Supplementary Table S1 and Supplementary Figure S1) and collected fresh stool samples. At the end of the experiment, all male mice were euthanized (CO_2_ inhalation after anesthesia with halothane) for organ, blood, and spermatozoa collection.

### 2.3. Blood, Organs, and Sperm Samples

Immediately following euthanasia, blood was collected via cardiac puncture and transferred into Microvette^®^ capillary tubes (Sarstedt, Nümbrecht, Germany). Blood serum was separated by centrifugation at 2000× *g* for 10 min at 4 °C, then stored at −80 °C. Reproductive organs, including the testes, epididymides, seminal vesicles, and prostate, were excised and weighed. Tissues were subsequently either snap-frozen in liquid nitrogen and stored at −80 °C or fixed for histological analysis. Epididymal and testicular tissues were fixed in Modified Davidson Fixative (30% of 37–40% formalin, 15% ethanol, 5% glacial acetic acid, and 50% distilled H_2_O), whereas the seminal vesicles, prostate, and non-reproductive tissues were fixed in 10% formaldehyde.

Cauda epididymides were placed in phosphate-buffered saline (PBS; 1 mM KH_2_PO_4_, 10 mM Na_2_HPO_4_, 137 mM NaCl, 2.7 mM KCl, pH 7.0) and carefully pierced five times using a 25G needle to allow spermatozoa to swim out for 10 min at 37 °C. Fresh sperm aliquots were used to assess different parameters of sperm quality, while remaining aliquots were stored at −80 °C.

### 2.4. 16S rRNA Sequencing and Analysis

Bacterial DNA was extracted from fecal samples at the MI4 Microbial Genomics Platform at McGill University, using the DNeasy PowerSoil Kit according to the manufacturer’s instructions (Qiagen, Hilden, Germany). The full-length 16S rRNA gene was amplified by PCR and sequenced using 250 bp paired-end technology on an Illumina MiSeq platform. Sequencing, library preparation, and quality control were performed by the MI4 platform following standard protocols adapted from previously published methods. Raw sequencing data were processed using the QIIME2 platform, including demultiplexing, quality filtering, and denoising with the DADA2 plugin to generate amplicon sequence variants (ASVs). Taxonomic assignment was performed against the SILVA database.

Downstream data processing and analysis were performed using MicrobiomeAnalyst 2.0 [30]. Low-count features were filtered using a minimum count threshold of 4 and a minimum prevalence of 20% across samples. Low-variance features were filtered using the interquartile range (IQR) method, with 10% of features removed. Data were transformed using centred log-ratio (CLR) transformation. No rarefaction or data scaling was applied. Statistical significance was accepted at *p* ≤ 0.05, with false discovery rate (FDR) correction applied where appropriate. Alpha diversity was assessed using Chao1 and Shannon diversity indices. Group differences in alpha diversity were evaluated using Hutcheson’s *t*-test/one-way ANOVA. Beta diversity was calculated using Jensen–Shannon divergence and visualized by principal coordinate analysis (PCoA). Group differences in beta diversity were assessed using permutational multivariate analysis of variance (PERMANOVA). Differential abundance analysis was performed using the Mann–Whitney U test. For taxonomic composition summaries, ASVs were considered at the family level.

### 2.5. Fecal Short-Chain Fatty Acid Extraction

Fecal SCFAs were extracted using a modified version of the method by Zhao et al. [31]. Stools were resuspended in Milli-Q water and homogenized using a MP Biomedical FastPrep instrument for 1 min at 4.0 m/s. Then, the suspension was acidified with 5 M HCl to a final pH of 2.0, incubated, and centrifuged at 9600× *g* to separate the supernatant. The fecal supernatants were then spiked with 2-ethylbutyric acid to a final concentration of 1 mM. Extracted SCFA supernatants were stored in 2 mL glass inserts. SCFAs were detected by gas chromatography using a Thermo Trace 1310 system coupled to a flame ionization detector (ThermoScientific, Markham, ON, Canada) and a Thermo TG-WAXMS A GC column (30 m × 0.32 mm × 0.25 µm) (ThermoScientific, Markham, ON, Canada).

### 2.6. Fecal and Tissue Lipid Extraction

Frozen fecal and testis samples were freeze-dried using a Lyophilizer Lock 6 Freeze Dryer (Labconco, Kansas City, MO, USA) set at −100 °C and 0–100 µHg for 2 h. Dried samples were stored at −20 °C. Freeze-dried samples were transferred to CK14 Soft Tissue Homogenizing tubes (Bertin Technology, Montigny-le-Bretonneux, France) and homogenized in 300 µL of ice-cold methanol using a Bead Mill. Homogenization was performed three times for 30 s each. Briefly, 900 µL of cold MTBE was added to the homogenates and incubated at room temperature for 10 min on an end-to-end rotator. Then, 250 µL of high-performance liquid chromatography (HPLC)-grade water was added, and samples were placed on ice for 10 min. Samples were centrifuged at 21,110× *g* for 20 min at 4 °C. Following centrifugation, the upper organic phase and lower aqueous phase were transferred into clean glass vials. Samples were evaporated using a Savant SPD1010-115 SpeedVac Concentrator (Fisher Scientific, Waltham, MA, USA) for 4 h without heating. Dried lipid and metabolite extracts were stored at −20 °C. Procedural blanks were processed identically to experimental samples, but without biological material.

### 2.7. Serum Lipid Extraction

Lipids were extracted from serum samples following a modified protocol described by Matyash et al. [32]. Briefly, 100 µL of serum was diluted with 3000 µL of ice-cold HPLC-grade methanol and vortexed for 30 s. Then, 500 µL of MTBE was added, and the mixture was placed on an end-to-end rotator for 10 min at room temperature. After which, 120 µL of HPLC-grade water was added, and samples were incubated on ice for 10 min. Samples were centrifuged at 21,000× *g* for 20 min at 4 °C. Following centrifugation, the upper organic phase and lower aqueous phase were separated and transferred into glass vials. Both phases were evaporated using a Savant SPD1010-115 SpeedVac Concentrator for 4 h without heating. Dried lipid and metabolite extracts were stored at −20 °C. Procedural blanks were prepared without adding biological samples.

### 2.8. Untargeted Lipidome Analysis by Ultra-High-Performance Liquid Chromatography Coupled to Mass Spectrometry (UHPLC-MS/MS) and Data Analysis

Dried lipid and metabolite extracts were resuspended in 160 µL of 8:1 methanol:toluene (*v*/*v*) and 160 µL of 60% acetonitrile:water (*v*/*v*), respectively. Samples were centrifuged at 14,000× *g* for 15 min at 4 °C and transferred into MS vials (Agilent Technologies, Overland Park, KS, USA). For quality control (QC), a pooled QC sample was generated by combining 20 µL aliquots from each biological sample into a single MS vial. All samples were analyzed using UHPLC-MS/MS.

Extracts were separated by liquid chromatography using an Ultimate 3000 Liquid Chromatography (100 mm length × 2.1 mm internal diameter × 1.9 µm particle size; Thermo Fisher Scientific, Waltham, MA, USA) coupled to a Q-Exactive Orbitrap High-Resolution Mass Spectrometry (Thermo Fisher Scientific, Waltham, MA, USA), equipped with a heated electrospray ionization (HESI) source. Chromatographic separation was performed with a constant flow rate of 0.3 mL/min; the column temperature was set at 50 °C, and the injection volume was 3 µL. The gradient elution was composed of mobile phase A (0.1% formic acid in water) and mobile phase B (0.1% formic acid in acetonitrile). The gradient was as follows: an initial condition of 1% B (0 min), increased to 2% B (0–0.5 min), to 4% B (0.5–0.9 min), 6% B (0.9–1.3 min), to 21% B (1.3–1.4 min), to a final condition of 99% B (1.4–11 min). The column was returned to initial conditions of 1% B (11–12 min). The mass spectrometer was operated in positive ion mode with a scan range of *m*/*z* 150–2000. Source parameters were set as follows: sheath gas flow rate, 5 au.; aux gas flow rate, 1 au.; spray voltage, +3.2 kV; capillary temperature, 320 °C; S-lens RF level, 50. For QC, biological samples were injected randomly; the pooled QC sample was used to equilibrate the system, and injections of both blanks and pooled QC samples were included between each set of five samples.

Raw mass spectral files were converted to mzML format using MSConvert and analyzed using MetaboAnalyst 6.0 [33]. Low-quality peaks were filtered using a sample-to-blank ratio threshold of 10, exclusion of features with greater than 50% missing values, and low-repeatability filtering using a relative standard deviation (RSD) threshold greater than 20%. Data were log10-transformed before downstream statistical analyses, and no data scaling was applied.

### 2.9. Assessment of Sperm Motility and Cytoplasmic Droplet Retention

Sperm motility parameters were evaluated using the HT-IVOS II computer-assisted semen analysis system (CASA) with the HT Morph Software version 1.0 (Hamilton Thorne, Beverly, MA, USA). Briefly, 5 µL of sperm samples were mixed with 95 µL of PBS containing bovine serum albumin (BSA; 5 mg/mL; Sigma-Aldrich, St. Louis, MO, USA), and 30 µL of the solution was loaded onto chamber slides (Leja, Nieuw-Vennep, The Netherlands) before being placed into the CASA system maintained at 37 °C. A minimum of 200 spermatozoa per sample were analyzed to assess total motility, progressive motility, hyperactivated motility, and velocity parameters.

Cytoplasmic droplet retention was evaluated according to previously established methods [34]. The percentage of spermatozoa exhibiting a visible cytoplasmic droplet was recorded.

### 2.10. Assessment of Acrosomal Integrity

Briefly, 10 µL of sperm suspension was smeared onto Superfrost Plus slides (Fisher Scientific, Nepean, ON, Canada) and incubated with Giemsa stain diluted 1:10 in distilled H_2_O (ddH_2_O) for 1.5 h at room temperature. After washing the slides with ddH_2_O, 30 µL of ddH_2_O was added onto each slide before placing a coverslip (Globe Scientific, Mahwah, NJ, USA). The percentage of spermatozoa with intact (Giemsa-stain-positive) and reacted (Giemsa-stain-negative) acrosomes of 200 spermatozoa per sample was determined using a Leica DMI6000 B inverted light microscope (Leica Microsystems, Concord, ON, Canada) at 40× magnification.

### 2.11. Histological Examination of Seminiferous Tubules

After fixation in modified Davidson fixative for 24 h at room temperature, testes were processed following standard formalin-fixed paraffin-embedded (FFPE) procedures, including graded ethanol dehydration, xylene clearing, paraffin infiltration, and paraffin embedding. Tissue sections were cut at 5 µm and stained with hematoxylin and eosin (H&E) or periodic acid–Schiff (PAS). The spermatogenesis stages and the number of spermatogonia and preleptotene spermatocytes (present at the level of the basal membrane of the seminiferous tubules), round spermatids and Sertoli cells were determined in ~200 seminiferous tubules at stage VII by analysis of PAS-stained testis sections per mice [35].

### 2.12. Immunocytochemical Detection of Sperm DNA Oxidation

We used 8-OHdG levels to assess DNA oxidation in spermatozoa. Briefly, 10 µL of sperm suspension was smeared onto Superfrost Plus slides. Slides were incubated in resuspension buffer (50 mM Tris-HCl, pH 7.4, 1% SDS, 40 nM DTT, and 1 mM EDTA) for 5 min at room temperature. Following incubation, slides were washed with phosphate-buffered saline (PBS; Fisher Bioreagents, Ottawa, ON, Canada). Slides were blocked with 5% goat serum (ThermoFisher Scientific, Markham, ON, Canada) in PBS for 1 h at room temperature. After washing with PBS, slides were incubated overnight at 4 °C with anti-8-OHdG FITC antibody (1:100). The following day, slides were washed with PBS and mounted using ProLong Gold Antifade with DAPI (ThermoFisher Scientific, Markham, ON, Canada) before applying coverslips. Negative control slides were incubated with PBS in place of the antibody. Fluorescent images were captured at identical exposure settings using a Leica DMI6000 inverted microscope at 40× magnification. FIJI ImageJ software (version 2.1.0/1.53c, National Institutes of Health, Stapleton, NY, USA) was used for background fluorescence subtraction and quantification of the average relative fluorescence intensity (RFI) of 200 spermatozoa per sample. DNA oxidation was expressed as the percentage of spermatozoa exhibiting strong 8-OHdG fluorescence.

### 2.13. Immunocytochemical Detection of Oxidative and Nitrosative Stress in Spermatozoa

To assess levels of oxidative and nitrosative stress-associated proteins, sperm samples were smeared onto Superfrost Plus slides. Depending on the protein of interest, slides were permeabilized in methanol for 10 min at −20 °C (PRDX6-SO_3_, iNOS, SOD1). Slides were then rehydrated in PBS for 5 min at room temperature. Slides were blocked for 30 min at room temperature with either 1% horse serum (for 4-HNE; MP Biomedicals, Solon, OH, USA) or 5% goat serum (for PRDX6-SO_3_, iNOS, nitrotyrosine and SOD1). After PBS washes, slides were incubated overnight at 4 °C with the appropriate primary antibodies: anti-4-HNE (1:100), anti-PRDX6-SO_3_ (1:100), anti-iNOS (1:100), anti-nitrotyrosine (1:100), and anti-SOD1 (1:100). Following PBS washes, slides were incubated for 1 h at room temperature with the appropriate secondary antibodies: goat anti-mouse (1:1000) for nitrotyrosine and iNOS; donkey anti-goat (1:2000) for 4-HNE; and goat anti-rabbit (1:1000) for SOD1 and PRDX6-SO_3_. ProLong Antifade mounting medium containing DAPI was applied, and coverslips were mounted. Negative control slides were incubated with secondary antibody alone. Fluorescence images were acquired using a Leica DMI6000 inverted microscope at 40× magnification with identical exposure settings for all samples. Image analysis was performed using FIJI (ImageJ) to subtract background fluorescence and quantify RFI. More than 200 spermatozoa were analyzed per sample. Protein fluorescence levels were expressed as arbitrary units (a.u.).

### 2.14. Assessment of Testosterone and Estradiol in Serum

Sera from ND-fed and HFD-fed males were used to determine total testosterone and estradiol by competitive enzyme-linked immunosorbent assay (Elabscience, Houston, TX, USA) according to the manufacturer’s instructions.

### 2.15. Assessment of Sperm Capacitation

To assess the fertilizing ability of spermatozoa, we incubated cauda epididymal spermatozoa in Biggers, Whitten and Wittingham (BWW) medium, with or without capacitation inducers (5 mg/mL BSA + 25 mM HCO_3_), for 60 min at 37 °C. Samples were re-centrifuged at 600× *g* for 5 min at 20 °C. The supernatant was discarded, then samples were incubated in fresh BWW with 10 µM progesterone for 30 min at 37 °C to promote the acrosome reaction as previously described [36]. Samples were then fixed with 4% paraformaldehyde for 15 min at 20 °C, centrifuged at 350× *g* for 5 min at 20 °C, and the pellet was resuspended in BWW. Samples were smeared onto Superfrost slides, air-dried, then incubated with 1:10 Giemsa stain diluted with distilled water for 1.5 h. After washing, slides were covered with a coverslip, and the percentage of spermatozoa with intact and reacted acrosomes was determined from 200 spermatozoa per sample using a Leica DM500 microscope (Opti-Tech Scientific, Montreal, QC, Canada) at 600× magnification. Sperm capacitation was determined as the percentage of spermatozoa that underwent progesterone-induced acrosome reaction, defined as the percentage of Giemsa-stain-negative spermatozoa.

### 2.16. Statistical Analysis

Results are presented as mean ± SEM. Statistical significance was accepted at *p* ≤ 0.05 and false discovery rate (FDR) < 0.05, unless stated otherwise. Statistical analyses were performed using GraphPad Prism version 10 (GraphPad Software Inc., San Diego, CA, USA). Normality and homogeneity of variance were assessed using the Shapiro–Wilk and Levene’s tests, respectively; for comparisons between two groups, two-tailed unpaired Student’s t-tests were used. For analyses involving multiple groups or variables, two-way ANOVA followed by Bonferroni post hoc correction was performed. Analyses included principal coordinate analysis (PCoA), volcano plot analysis, fold-change analysis, and Student’s t-tests were done using MetaboAnalyst version 6.0 [33]. For volcano plot analysis, significantly altered features were defined as those with a fold-change threshold of 1.5 and FDR adjusted *p* ≤ 0.05.

## 3. Results

### 3.1. High-Fat Diet Induces Gut Microbiota Dysbiosis

**Figure 1:**
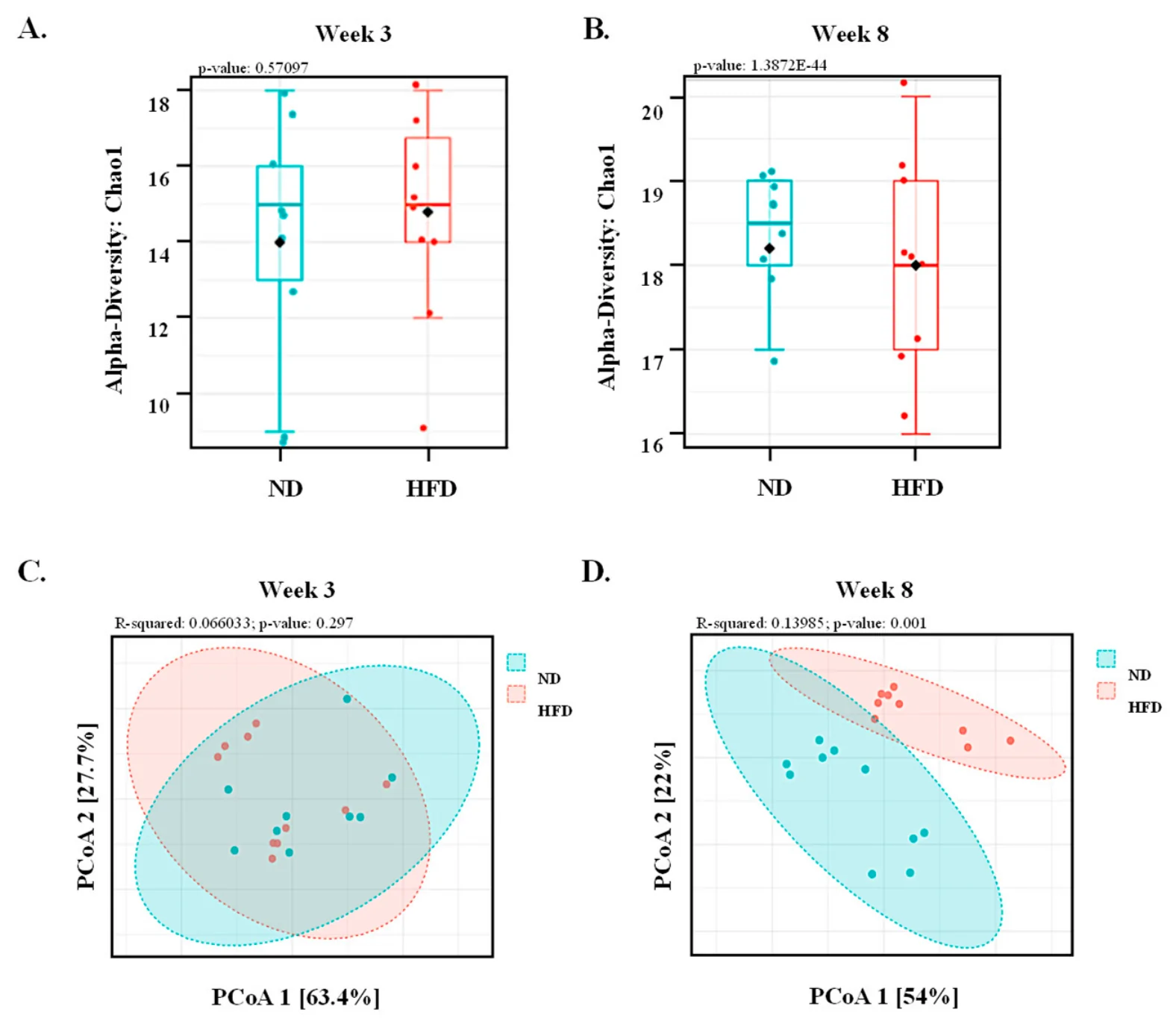
Alpha- and beta-diversity analyses of gut microbial community structure in normal diet (ND)-fed and high-fat diet (HFD)-fed mice. Stool samples from ND- and HFD-fed mice at 3 and 8 weeks of age were analyzed with 16S rRNA sequencing. (**A**,**B**) Alpha diversity was assessed using the Chao1 richness index. Box plots show the median, interquartile range (IQR), and minimum–maximum values. Statistical analysis was performed using the Hutcheson T-test and ANOVA. (**C**,**D**) Beta diversity was assessed using Bray–Curtis and visualized by principal coordinate analysis (PCoA). Statistical analysis was performed using PERMANOVA; *n* = 9–10 mice per group.

To assess the influence of a high-fat diet on gut microbial composition, we characterized changes in gut microbiota. We collected and analyzed stool samples from 3-week-old and 8-week-old ND-fed and HFD-fed mice. During the initial phase of the study (weeks 3 to 4), body weight did not differ significantly between ND-fed and HFD-fed mice (Figure S1). However, from weeks 5 to 8, HFD-fed mice had significantly higher body weights than ND-fed mice. We assessed alpha diversity (within-sample microbial richness and evenness) and beta diversity (between-sample differences in microbial community composition) to evaluate microbial diversity and community structure. As expected, at 3 weeks of age, we observed no significant differences between ND-fed and HFD-fed mice, as alpha diversity metrics (Chao1 index) showed overlapping microbial richness between the two groups (Figure 1A). However, by 8 weeks, HFD-fed mice showed a significant reduction in alpha diversity compared with ND-fed controls, indicating a loss of microbial diversity (Figure 1B). Consistent with these findings, beta-diversity analysis demonstrated that microbial community composition diverged between ND-fed and HFD-fed mice by the study endpoint (8 weeks of age) compared with the beginning of the study at 3 weeks (Figure 1C,D). Weighted principal coordinate analysis (PCoA) revealed clear separation between the microbial communities from both groups, with a significant difference along the PCoA1 axis, which accounted for 54% of the total variation (Figure 1D).

**Figure 2:**
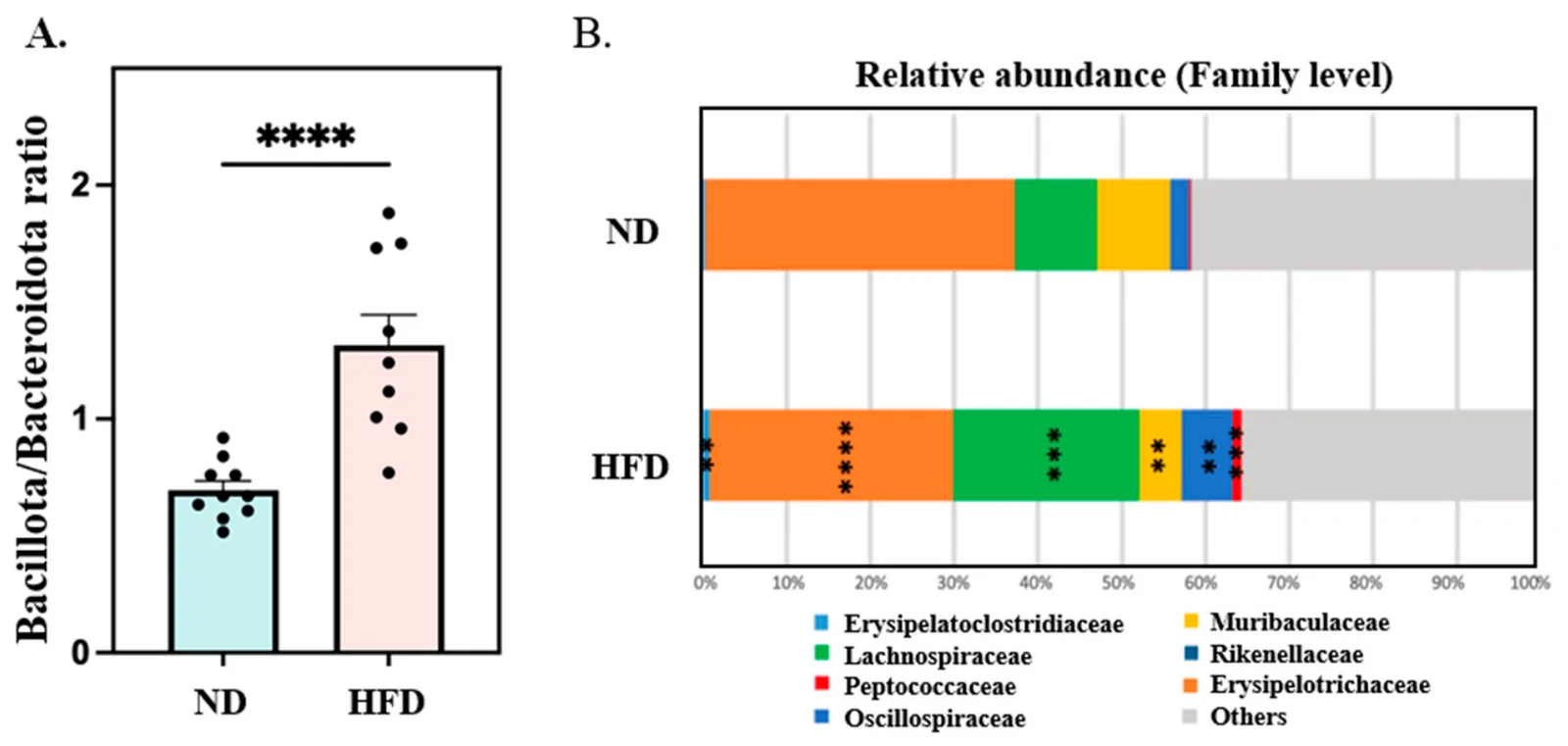
High-fat diet (HFD) alters gut microbiota composition. Stool samples from normal diet (ND)- and HFD-fed mice at 8 weeks of age were analyzed with 16S rRNA sequencing. (**A**) The relative abundance ratio of Bacillota to Bacteroidota. Statistical analysis was performed using Student’s *t*-test. Data are expressed as mean  ±  SEM. (**B**) The relative abundance of bacterial families. Statistical analysis was performed using the Mann–Whitney U test. *n* = 10 (ND) and 9 (HFD) male mice. ** *p* ≤ 0.01, *** *p* ≤ 0.001, **** *p* ≤ 0.0001.

The Bacillota-to-Bacteroidota ratio was 1.9-fold significantly higher in HFD-fed mice than in ND-fed controls (Figure 2A). This increase was accompanied by a higher relative abundance of Bacillota and a lower relative abundance of Bacteroidota (Supplementary Figure S2A). At the family level, the relative abundances of *Erysipelatoclostridiaceae, Lachnospiraceae, Peptococcaceae,* and *Oscillospiraceae* were significantly increased by 2.0-, 2.4-, 2-, and 2.8-fold, respectively, in HFD-fed mice (Figure 2B). In contrast, the relative abundances of *Muribaculaceae, Rikenellaceae* and *Erysipelotrichaceae* were significantly decreased by 2.5-, 3.3-, and 1.3-fold, respectively, in HFD-fed mice compared to ND-fed controls (Figure 2B). At the genus level, HFD feeding was associated with a 2.3-fold decrease in *Dubosiella* abundance and a 1.8-fold increase in *Akkermansia* abundance (Supplementary Figure S2B).

### 3.2. High-Fat Diet Alters Short-Chain Fatty Acid Levels

**Figure 3:**
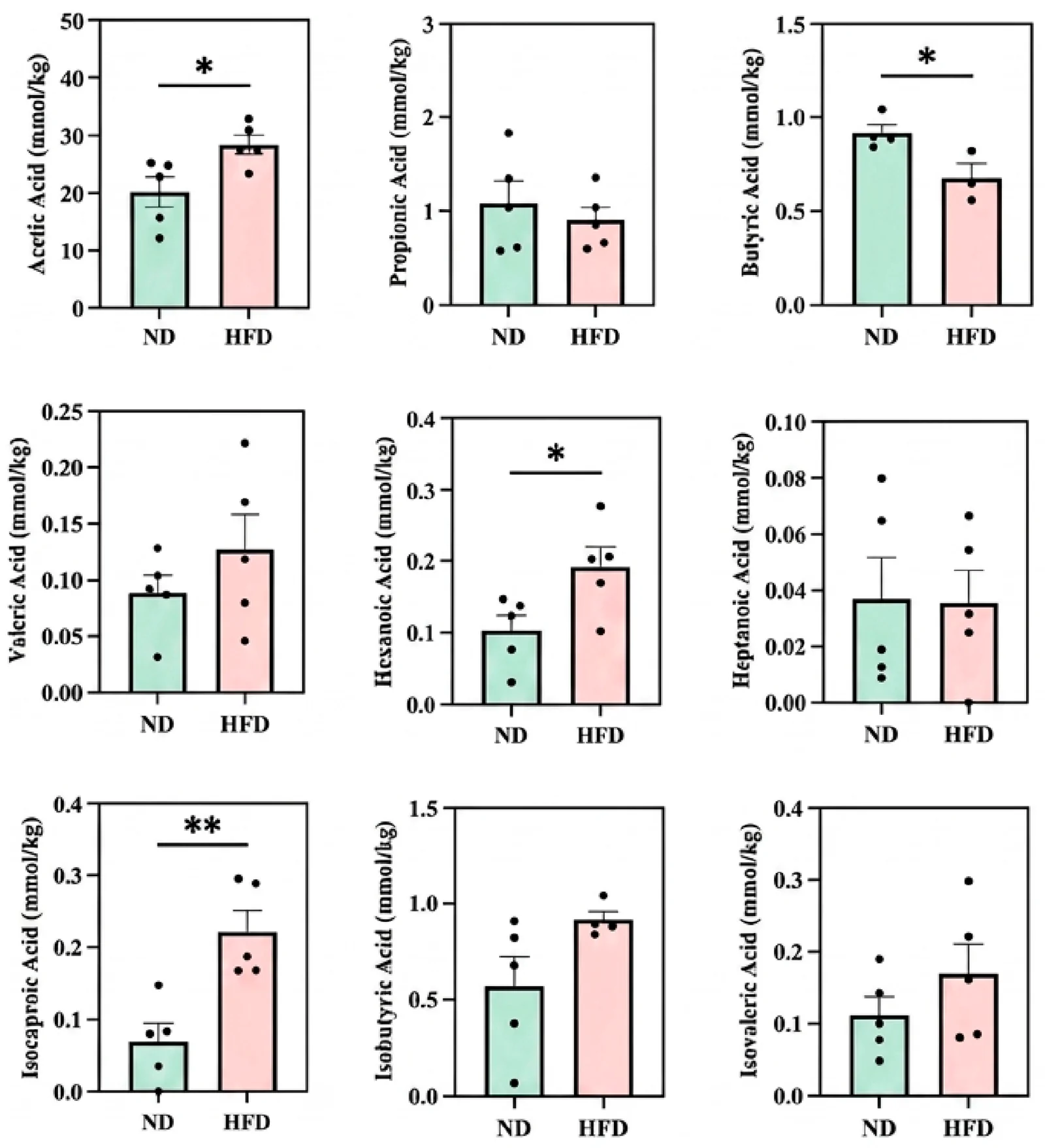
Short-chain fatty acid (SCFA) concentration levels in normal diet (ND)-fed and high-fat diet (HFD)-fed mice. Levels of SCFAs, including acetic acid, propionic acid, butyric acid, valeric acid, hexanoic acid, heptanoic acid, isocaproic acid, isobutyric acid and isovaleric acid, were measured in stool samples collected from 8-week-old ND-fed and HFD-fed mice. The concentrations of individual SCFAs were quantified and compared between groups. Data are expressed as mean  ±  SEM. Statistical significance was determined using Student’s *t*-test. *n* = 5 male mice per group. * *p* ≤ 0.05, ** *p* ≤ 0.01.

To determine whether HFD-induced gut microbial dysbiosis alters microbial metabolite production, we measured short-chain fatty acid (SCFA) levels, a major class of gut-microbiota-derived metabolites, in stool samples from 8-week-old ND-fed and HFD-fed mice. Quantitative analysis revealed that HFD-fed mice had significantly increased levels of acetic acid, hexanoic acid, and isocaproic acid (1.4-, 1.8-, 3.2-fold, respectively), and a significant 1.4-fold reduction in butyric acid compared to ND controls (Figure 3). No significant differences were observed between groups for propionic acid, valeric acid, heptanoic acid, isobutyric acid, or isovaleric acid. Although not statistically significant, propionic acid and heptanoic acid were lower in HFD-fed mice than in ND-fed mice. In contrast, valeric acid, isobutyric acid, and isovaleric acid exhibited an increasing trend in HFD-fed mice relative to ND controls (Figure 3).

### 3.3. High-Fat Diet Promotes Distinct Stool, Serum and Testis Lipidomic Remodelling with Enrichment of Sphingolipid Species

**Figure 4:**
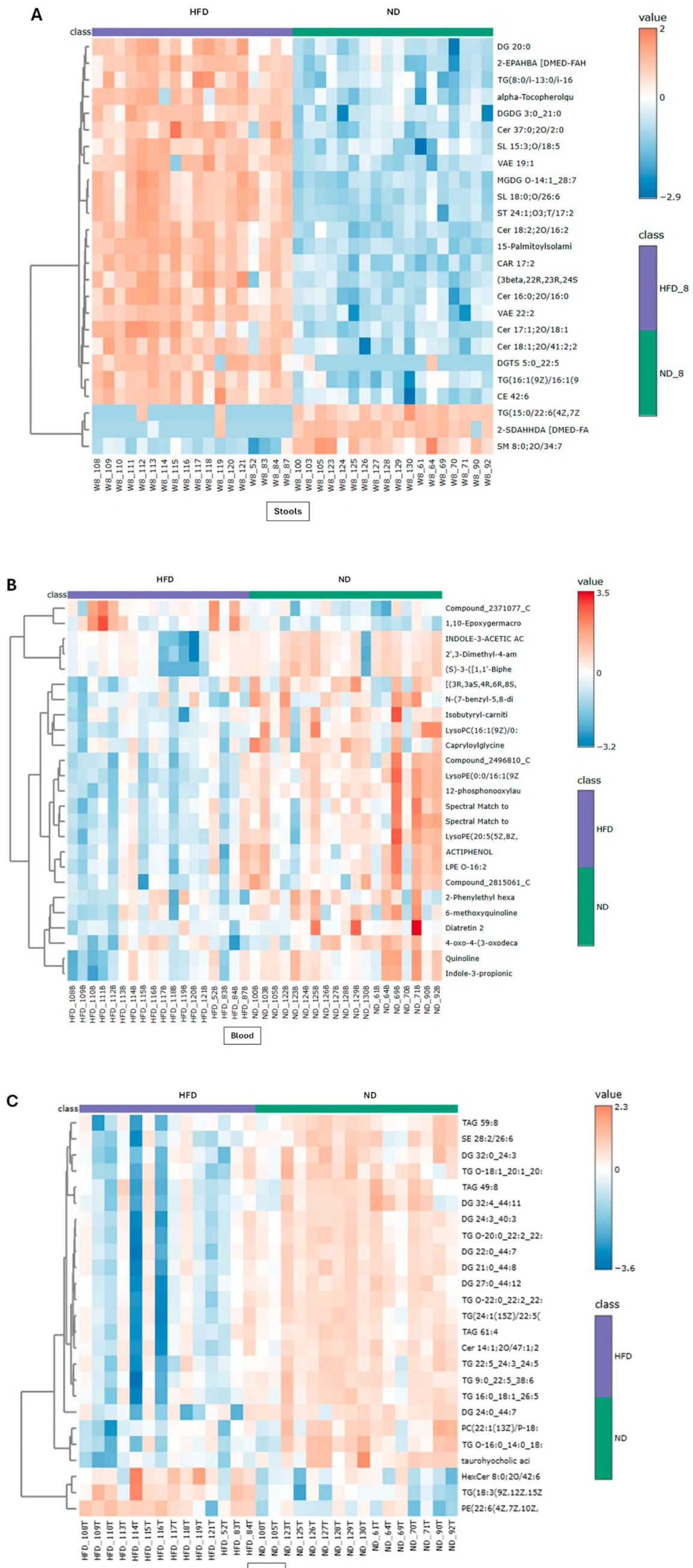
Hierarchical clustering heat maps of differentially abundant lipids in stool, serum, and testis samples from normal diet (ND)- and high-fat diet (HFD)-fed mice. Heat maps show the top lipid species identified as differentially abundant between ND- and HFD-fed mice by ANOVA (*p* ≤ 0.05). Data obtained by UHPLC–MS/MS were processed, normalized, and visualized using MetaboAnalyst 6.0. (**A**) Stool, (**B**) serum, and (**C**) testis lipid profiles. Rows represent individual lipid species, and columns represent individual mouse samples from the ND (*n* = 19) and HFD (*n* = 18) groups. The colour scale represents scaled relative lipid abundance, ranging from lower (blue) to higher (red) abundance relative to the mean abundance of each lipid across samples. Hierarchical clustering was performed using Euclidean distance and Ward’s linkage method.

We next investigated whether HFD-induced gut microbiota dysbiosis was accompanied by alterations in the lipid profiles of stool, serum, and testis samples. Unsupervised hierarchical clustering heat maps revealed a clear metabolic divergence between HFD- and ND-fed mice (Figure 4). Across the three sample types, lipid species—the majority of which were sphingolipids—were elevated in abundance in HFD-fed mice and generally showed lower abundance in ND-fed mice when compared between these groups. Lipid species reduced in HFD samples—the majority of which were triglycerides—were correspondingly higher in ND controls, demonstrating an inverse abundance pattern between the two dietary groups.

### 3.4. High-Fat Diet Increases Sphingolipid-Producing Gut Bacteria in Stool Samples

**Figure 5:**
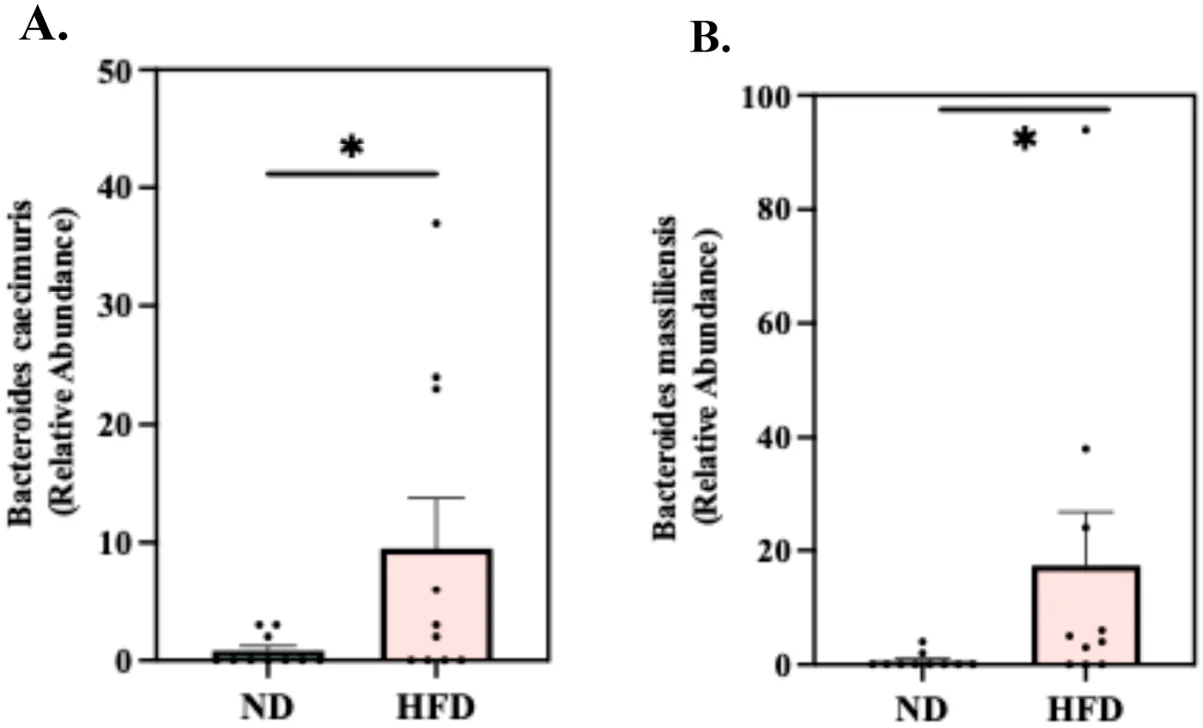
High-fat diet (HFD) increases sphingolipid-producing gut bacteria in stool samples. Relative abundance of gut microbiota reveals increased levels of sphingolipid-producing bacterial species in stool samples of 8-week-old HFD-fed mice compared to ND-fed mice. (**A**) Bacteroides caecimuris and (**B**) Bacteroides massiliensis. Data are expressed as mean  ±  SEM. Statistical significance was assessed using Student’s *t*-test. *n* = 6–10 male mice per group. * *p* ≤ 0.05.

To further characterize gut microbial dysbiosis associated with lipidomic remodelling, we analyzed stool samples from 8-week-old mice and found that HFD increased the relative abundance of sphingolipid-producing bacterial species 10-fold. Bacteroides caecimuris was significantly enriched in HFD-fed mice compared to the ND-fed mice (Figure 5A). Bacteroides massiliensis abundance increased 28-fold in HFD-fed mice compared with ND-fed mice (Figure 5B). ND-fed mice exhibited little-to-no detectable abundance of both bacterial species.

### 3.5. High-Fat Diet Alters Gut Microbial Sphingolipid Profile in Stool Samples

**Figure 6:**
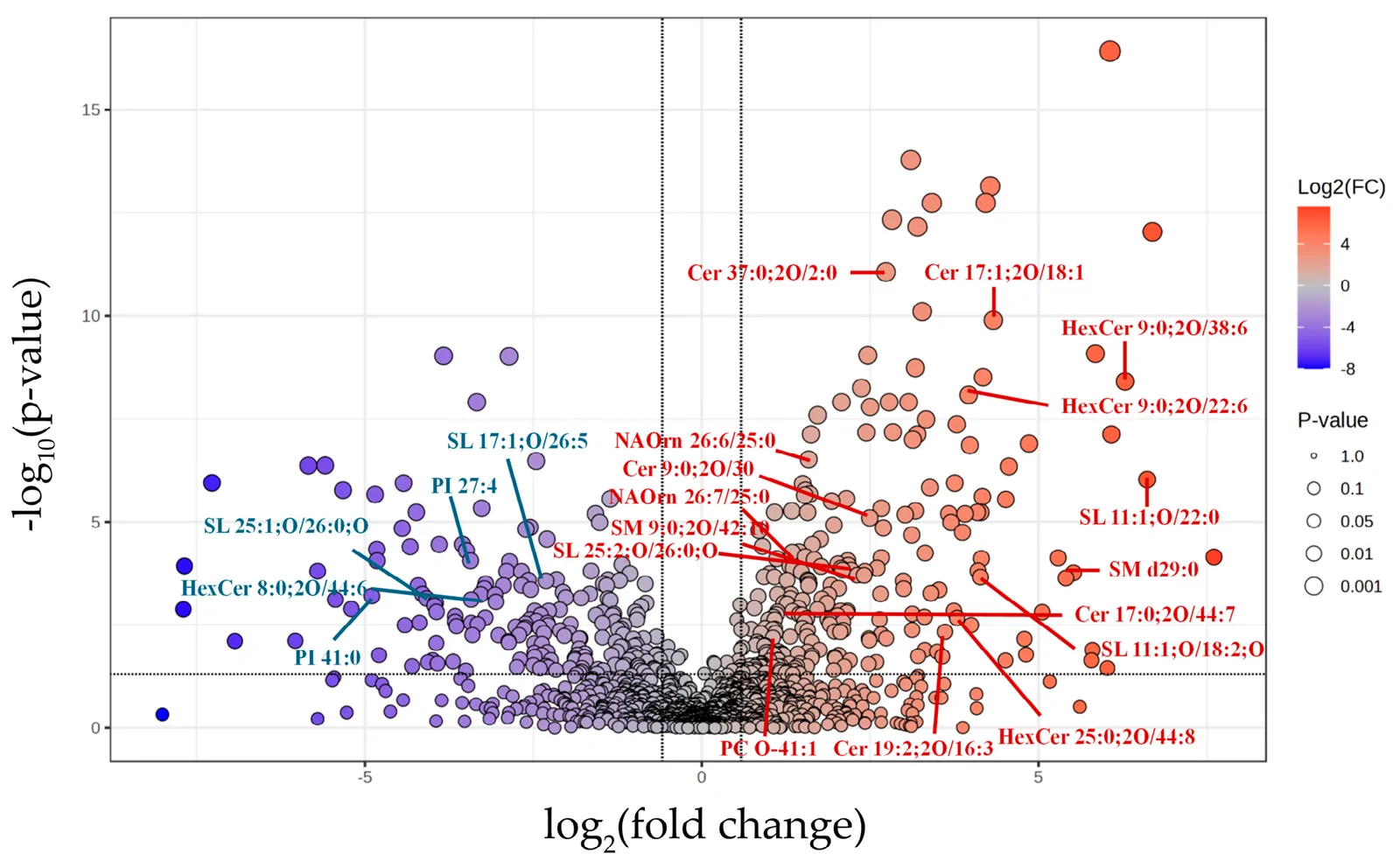
Volcano plot of stool lipid species in high-fat diet (HFD)-fed versus normal diet (ND)-fed male mice. Stool samples from 8-week-old ND- and HFD-fed mice were analyzed by UHPLC–MS/MS. The volcano plot shows the log_2_ fold change (log_2_FC; HFD relative to ND) on the *x*-axis and the −log_10_-transformed *p*-value obtained using a two-sample *t*-test on the *y*-axis. Positive log_2_FC values indicate higher relative abundance in HFD-fed mice, whereas negative values indicate lower relative abundance. The horizontal solid line indicates the statistical significance threshold (*p* ≤ 0.05). Vertical dotted lines indicate the applied fold-change threshold. Point colour represents the magnitude and direction of the log_2_FC, ranging from decreased abundance (blue) to increased abundance (red), and point size represents the *p*-value. *n* = 19 ND and 18 HFD male mice. Selected putatively microbiota-associated lipids are labelled, including N-acyl ornithine lipids (NAOrn), ceramides (Cer), sphingolipids (SL), sphingomyelins (SM), hexosylceramides (HexCer), phosphatidylcholines (PC), and phosphatidylinositols (PI).

We generated a volcano plot to visualize differences in stool lipid species between HFD- and ND-fed mice (Figure 6). We detected 9037 lipid species, of which 842 were annotated. Among the annotated lipids, 239 differed significantly between dietary groups: 113 showed increased relative abundance, and 126 showed decreased relative abundance in HFD-fed mice compared with ND-fed controls. Selected lipid classes of potential microbial relevance, including N-acyl ornithine lipids (NAOrn), ceramides (Cer), sphingolipids (SL), sphingomyelins (SM), hexosylceramides (HexCer), phosphatidylcholines (PC), and phosphatidylinositols (PI), were highlighted in the volcano plot. Among 105 lipids classified as putatively microbiota-associated based on lipid class and/or structural characteristics, including the presence of odd-chain fatty acyl groups, 16 were significantly increased and five were significantly decreased in stool samples from HFD-fed mice. The remaining 85 putatively microbiota-associated lipids did not differ significantly between dietary groups.

### 3.6. High-Fat Diet Alters Gut Microbial Sphingolipid Profile in Serum

**Figure 7:**
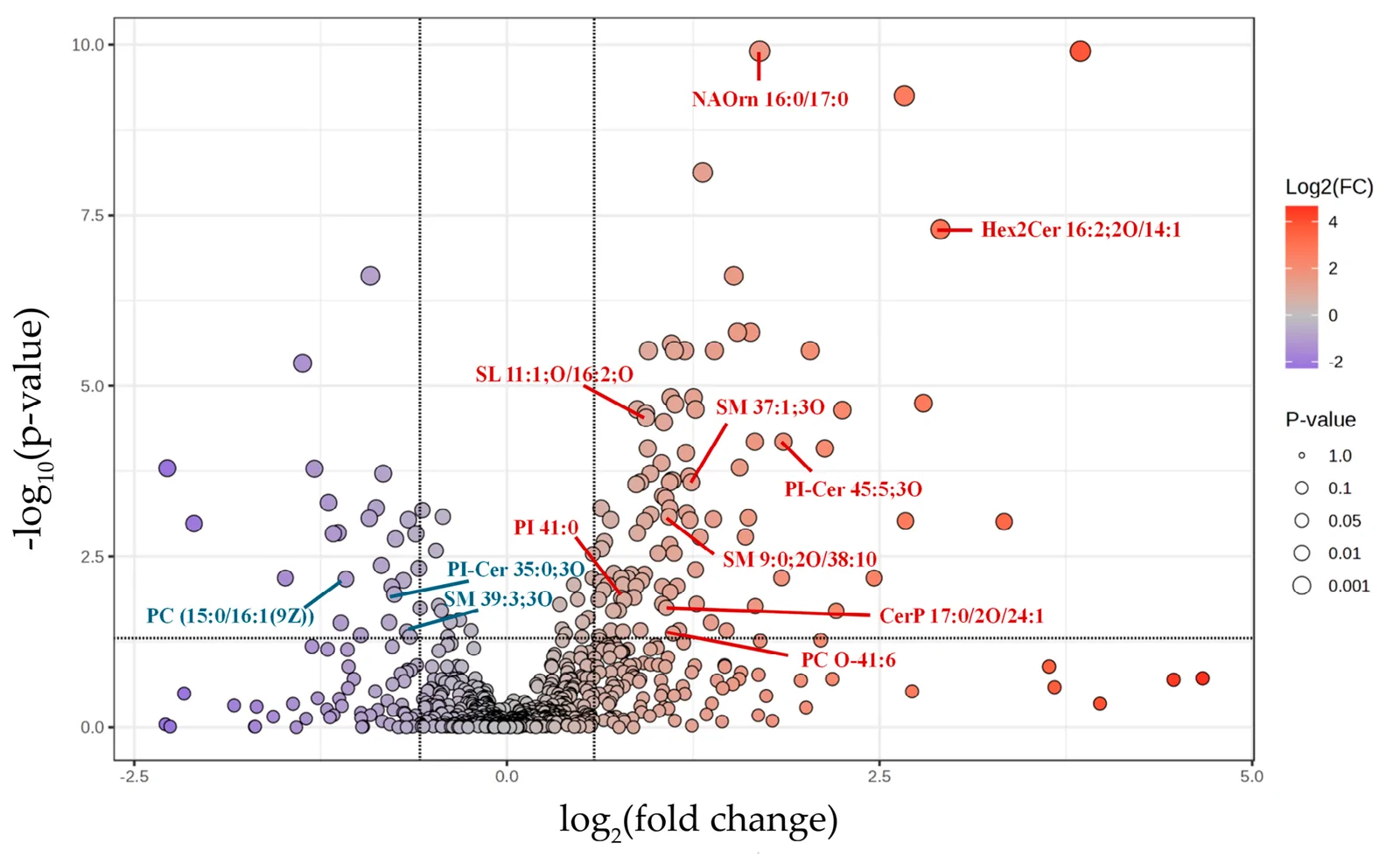
Volcano plot of serum lipid species in high-fat diet (HFD)-fed versus normal diet (ND)-fed mice. Serum samples from 8-week-old ND- and HFD-fed mice were analyzed using UHPLC–MS/MS-based lipidomics. The volcano plot shows the log_2_ fold change (log_2_FC; HFD relative to ND) on the *x*-axis and the −log_10_-transformed *p*-value obtained using a two-sample *t*-test on the *y*-axis. Positive log_2_FC values indicate higher relative abundance in HFD-fed mice, whereas negative values indicate lower relative abundance. The horizontal solid line indicates the statistical significance threshold (*p* ≤ 0.05), and the vertical dotted lines indicate the applied fold-change threshold. Point colour represents the magnitude and direction of the log_2_FC, ranging from decreased abundance (blue) to increased abundance (red), while point size represents the *p*-value. *n* = 16 male mice per group. Selected putatively microbiota-associated lipids are labelled.

Given the pronounced alterations observed in the stool lipid profile, we next investigated whether HFD consumption was also associated with changes in circulating lipids. Serum samples from 8-week-old ND- and HFD-fed mice were analyzed using UHPLC–MS/MS-based lipidomics. We detected 7117 lipid species, of which 804 were annotated. Among the annotated lipids, 77 differed significantly between dietary groups: 53 showed increased relative abundance, and 24 showed decreased relative abundance in HFD-fed mice compared with ND-fed controls (Figure 7). In addition, 19 lipids were classified as putatively microbiota-associated based on their lipid class and/or structural characteristics. Of these, nine were significantly increased, and three were significantly decreased in HFD-fed mice, whereas the remaining seven did not differ significantly between dietary groups. These findings indicate that HFD consumption was associated with alterations in the circulating lipid profile, including changes in several lipids of potential microbial relevance.

### 3.7. High-Fat Diet Alters Gut Microbial Sphingolipid Profile in Testis

**Figure 8:**
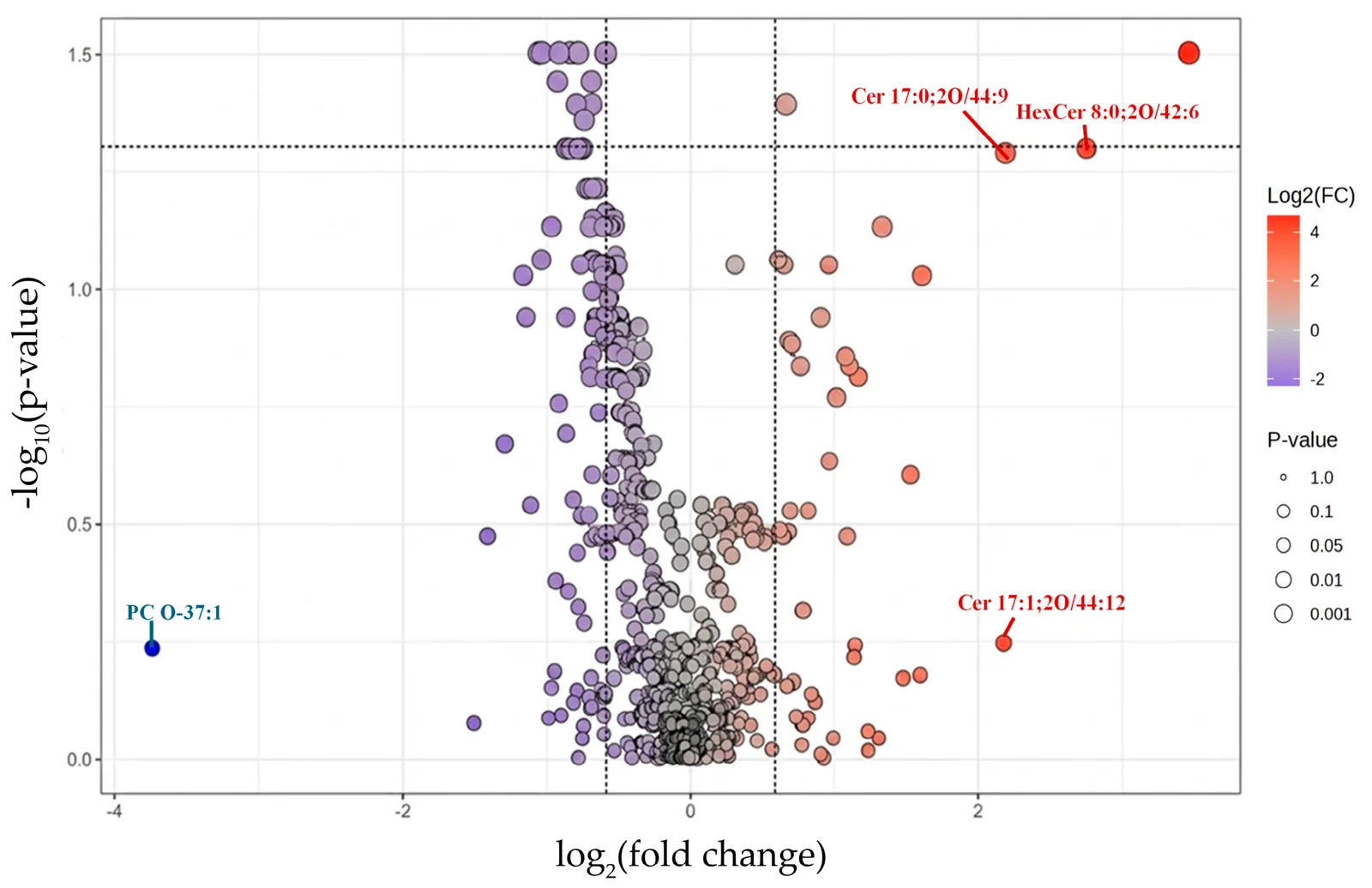
Volcano plot of testicular lipid species in high-fat diet (HFD)-fed versus normal diet (ND)-fed mice. Testis samples from 8-week-old ND- and HFD-fed mice were analyzed using UHPLC–MS/MS-based lipidomics. The volcano plot shows the log_2_ fold change (log_2_FC; HFD relative to ND) on the *x*-axis and the −log_10_-transformed *p*-value obtained using a two-sample *t*-test on the *y*-axis. Positive log_2_FC values indicate higher relative abundance in HFD-fed mice, whereas negative values indicate lower relative abundance. The horizontal dotted line indicates the statistical significance threshold (*p* ≤ 0.05), and the vertical dotted lines indicate the applied fold-change threshold. Point colour represents the magnitude and direction of the log_2_FC, ranging from decreased abundance (blue) to increased abundance (red), while point size represents the *p*-value. *n* = 18 male mice per group. Selected putatively microbiota-associated lipids are labelled, irrespective of statistical significance.

Given the HFD-associated alterations observed in stool and serum lipid profiles, we next investigated whether HFD consumption was also associated with changes in the testicular lipid profile. Testis samples from 8-week-old ND- and HFD-fed mice were analyzed using UHPLC–MS/MS-based lipidomics. A total of 3627 lipid species were detected, of which 759 were annotated. Among the annotated lipids, 15 differed significantly between dietary groups: two showed increased relative abundance, and 13 showed decreased relative abundance in the testes of HFD-fed mice compared with ND-fed controls (Figure 8). The remaining 744 annotated lipids did not differ significantly between groups. Four lipids were classified as putatively microbiota-associated based on their lipid class and/or structural characteristics. Of these, two were significantly increased in HFD-fed mice (Cer 17:0;20O/44:9 and HeCer 8:0;2O/42:6), whereas the other two did not differ significantly between dietary groups. Although PC O-37:1 and Cer 17:1;2O/44:12 did not reach statistical significance, they exhibited large fold changes, with PC O-37:1 markedly decreased and Cer 17:1;2O/44:12 increased in HFD-fed mice. These pronounced changes may be biologically relevant and warrant further investigation in larger cohorts.

Overall, HFD consumption produced comparatively limited changes in the testicular lipid profile, although selected lipids of potential microbial relevance were increased.

### 3.8. High-Fat Diet Produced Structurally Related Lipids Are Shared Across Stool, Serum and Testis Samples

No identical individual lipid species were shared across all three sample types. However, several structurally related lipids with similar backbone compositions were detected in stool, serum and testis samples; these mainly included sphingolipid species such as Cer, CerP, HexCer, Hex2Cer, HexCer 8, SL, and SM, as well as various phospholipids, such as PC and PI (Table 1 and Figure S6). Interestingly, Ceramide 17 was shared across stool, serum, and testis; Hex2Cer 16, SM 9, SL 11, PC O-41 and PI 41:0 were shared between stool and serum; and one lipid species was shared between stool and testis. These lipids differed primarily in fatty acyl chain composition. Notably, ceramides, hydroxy-ceramides, sphingomyelin, and phosphatidylcholine species with shared backbones were significantly elevated in HFD-fed mice compared to ND-fed controls across all sample types. These findings suggest that although identical lipid species were not conserved across tissues, related lipids with similar backbones shared among stool, serum and/or testis may indicate gut-microbiota-driven modifications or systemic lipid processing.

### 3.9. Gut Microbiome–Lipidomic Correlation in High-Fat Diet-Fed Mice

To explore potential relationships between gut microbial composition and lipid metabolism, we correlated stool bacterial family abundance with selected lipid species detected in stool and serum samples (Figure S3). Correlations were calculated across the combined ND and HFD cohorts and visualized as clustered heat maps using MicrobiomeAnalyst (Figure S3). Therefore, the observed associations may reflect both relationships between bacterial taxa and lipid abundance and their shared responses to dietary treatment.

In stool samples, several putatively microbiota-associated sphingolipids displayed significant correlations with bacterial family abundance. SM 9:0;2O/42:1;O and SL 11:1;O/18:2;O were positively correlated with *Lachnospiraceae, Oscillospiraceae,* and *Peptococcaceae* and negatively correlated with *Muribaculaceae* (Figure S2A). Other selected lipids, including HexCer 17:0;3O/13:0;(2OH), HexCer 9:0;2O/38:6, SM 29:0;2O, and SL 17:1;O/26:5, were positively correlated with *Erysipelatoclostridiaceae* and negatively correlated with *Muribaculaceae*.

We also observed associations between stool bacterial family abundance and serum lipid species (Figure S3B). In particular, the serum sphingomyelins SM 37:1;3O and SM 39:1;2O were positively correlated with *Peptococcaceae*. Additional associations involved a bacterial feature designated “Not_Assigned,” indicating that the corresponding taxon could not be classified at the family level. Collectively, these results suggest coordinated changes in gut bacterial composition and lipid abundance in response to HFD consumption, although additional analyses controlling for dietary group are required to establish diet-independent microbiota–lipid associations.

### 3.10. High-Fat Diet Impairs Sperm Quality

**Figure 9:**
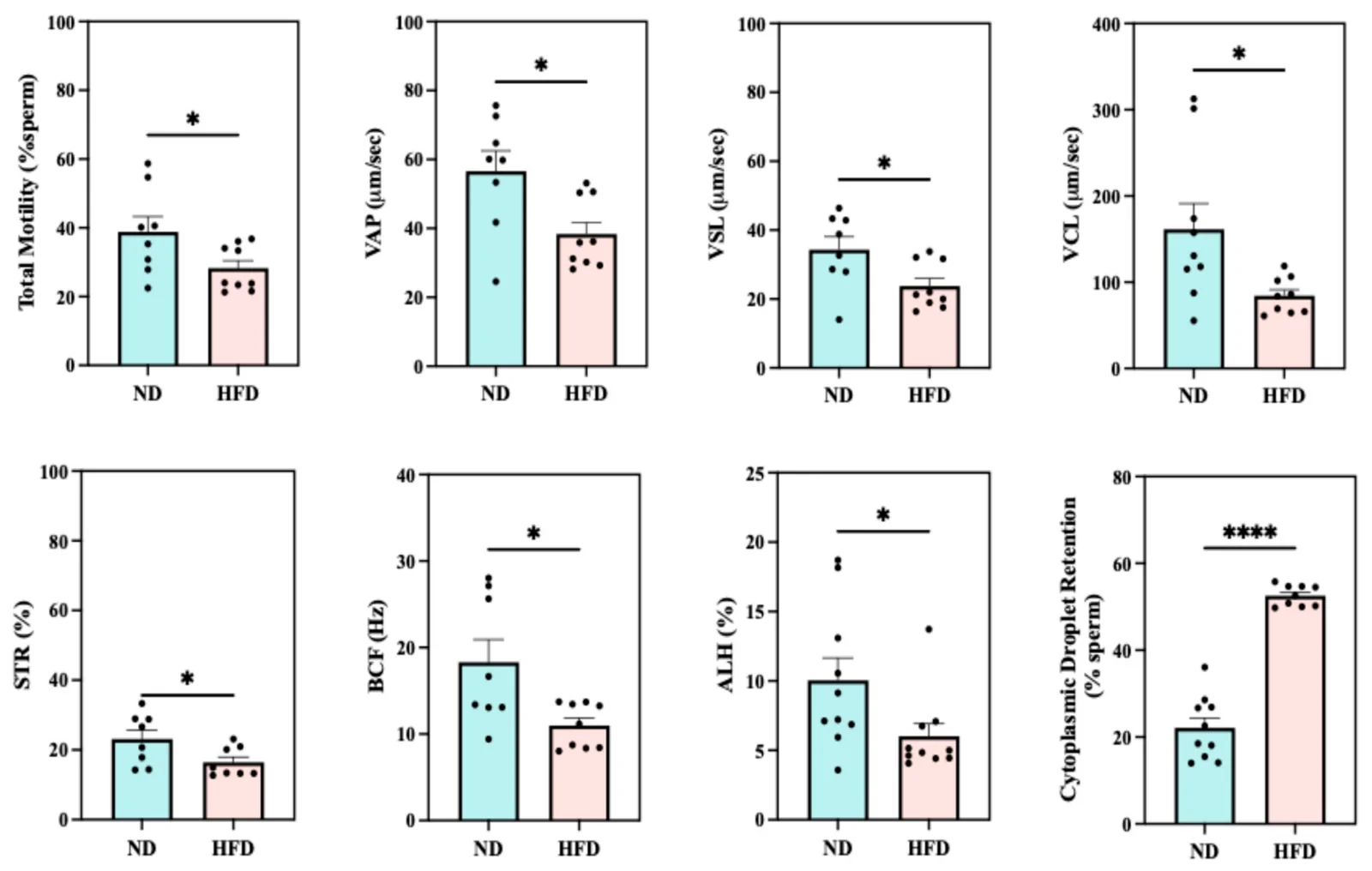
HFD impairs sperm motility parameters and cytoplasmic droplet retention. Sperm motility, average path velocity (VAP), linear velocity (VSL), curvilinear velocity (VCL), straightness (STR), beat cross frequency (BCF), and amplitude of lateral head displacement (ALH) in ND- and HFD-fed mice were analyzed by computer-assisted semen analysis. Percentage of spermatozoa displaying cytoplasmic droplet retention was determined using light microscopy. Data are expressed as mean  ±  SEM. More than 200 spermatozoa were evaluated per sample. Statistical analysis was performed using Student’s *t*-test. *n* = 8–10 male mice per group. * *p* ≤ 0.05 and **** *p* ≤ 0.0001.

We characterized the impact of HFD-induced gut microbial dysbiosis on sperm quality. First, we determined sperm motility and kinetics using a CASA system. All motility and kinematic parameters were significantly reduced (total motility 1.8-fold lower, VAP 1.4-fold lower, VSL 1.5-fold lower, VCL 2-fold lower, STR 1.1-fold lower, BCF 1.5-fold lower, and ALH 1.6-fold lower) in the HFD-fed group compared with ND-fed mice. We quantified cytoplasmic droplet retention (CDR), a marker of impaired sperm epididymal maturation, by bright-field microscopy. HFD-fed mice exhibited a significantly higher, 2.4-fold percentage increase in spermatozoa retaining cytoplasmic droplets compared to ND-fed controls (Figure 9).

**Figure 10:**
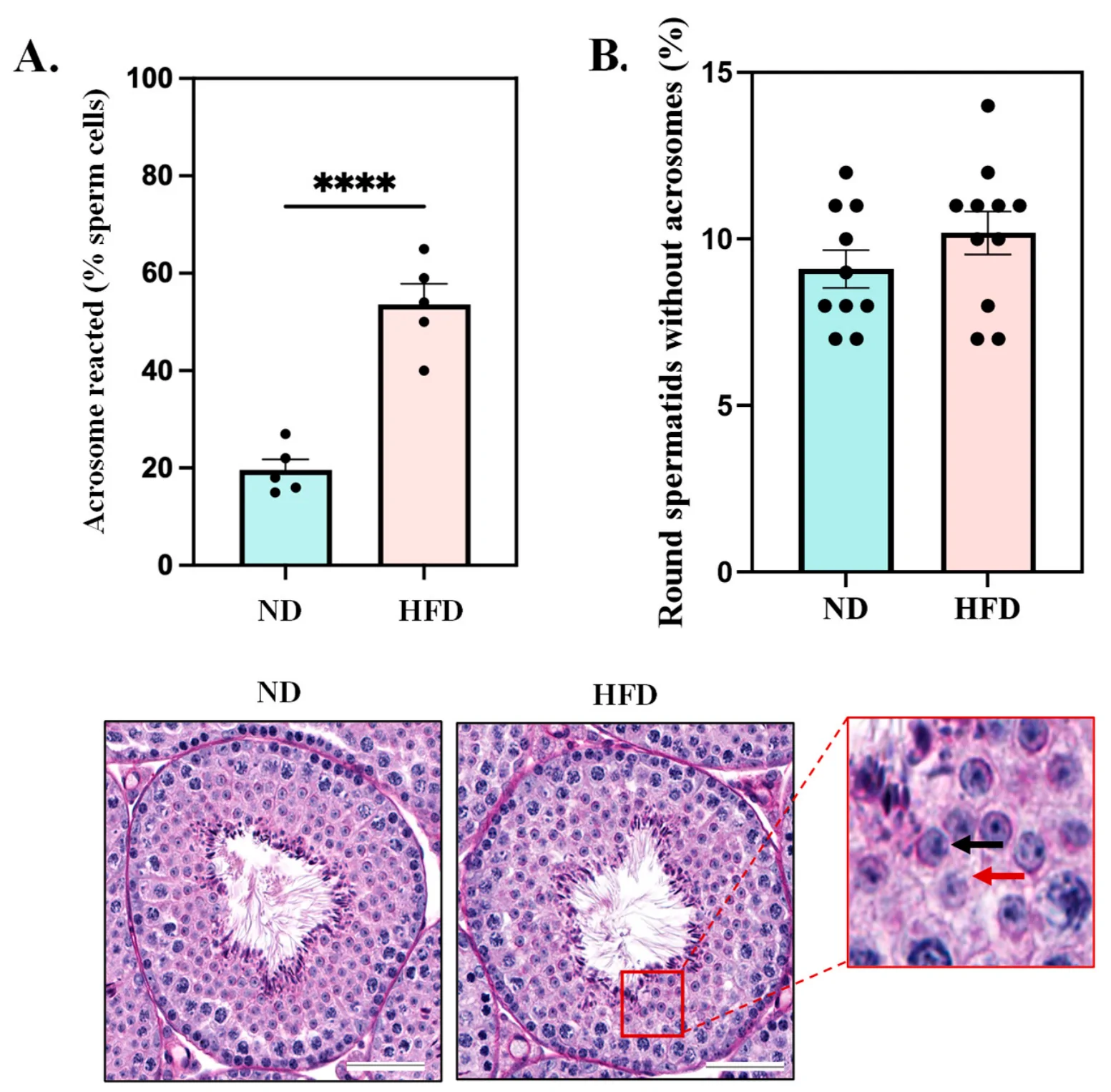
Acrosomal integrity is reduced in spermatozoa from high-fat diet (HFD)-fed males. (**A**) Quantification of the percentage of acrosome-reacted spermatozoa (spontaneous acrosome reaction) in ND and HFD mice (*n* = 5 male mice per group). More than 200 spermatozoa were evaluated per sample. Data are expressed as mean  ±  SEM. (**B**) Quantification of the percentage of round spermatids lacking acrosomes in ND and HFD mice testes. Below the graphs, representative images of PAS-stained seminiferous tubules at stage VII from ND-fed and HFD-fed mice illustrate germ cells with acrosomes (black arrow) and without acrosomes (red arrow). More than 200 tubules were scored for each group. *n* = 10 (ND) and 11 (HFD) male mice. Data are expressed as mean  ±  SEM. Statistical analysis was performed using Student’s *t*-test. **** *p* ≤ 0.0001. Scale bar = 50 µm.

To determine whether HFD affects acrosomal membrane integrity, we quantified the percentage of acrosome-reacted spermatozoa in ND- and HFD-fed mice. HFD-fed mice exhibited a significantly higher, 2.8-fold increase in the proportion of acrosome-reacted spermatozoa compared to ND controls (Figure 10A). Histological evaluation of the testes revealed no significant differences in germ cell development between ND and HFD testes, as all stages of spermatogenesis (I–XII) were present (Figure S4). The number of spermatogonia and preleptotene spermatocytes, round spermatids and Sertoli cells were similar in both groups (Table S2). However, we observed a small and significant difference in the testis weight (Figure S1B). The microscopic examination of H&E-stained sections did not reveal overt morphological abnormalities in the prostate or seminal vesicles.

### 3.11. High-Fat Diet Increases Oxidative Stress in Spermatozoa

**Figure 11:**
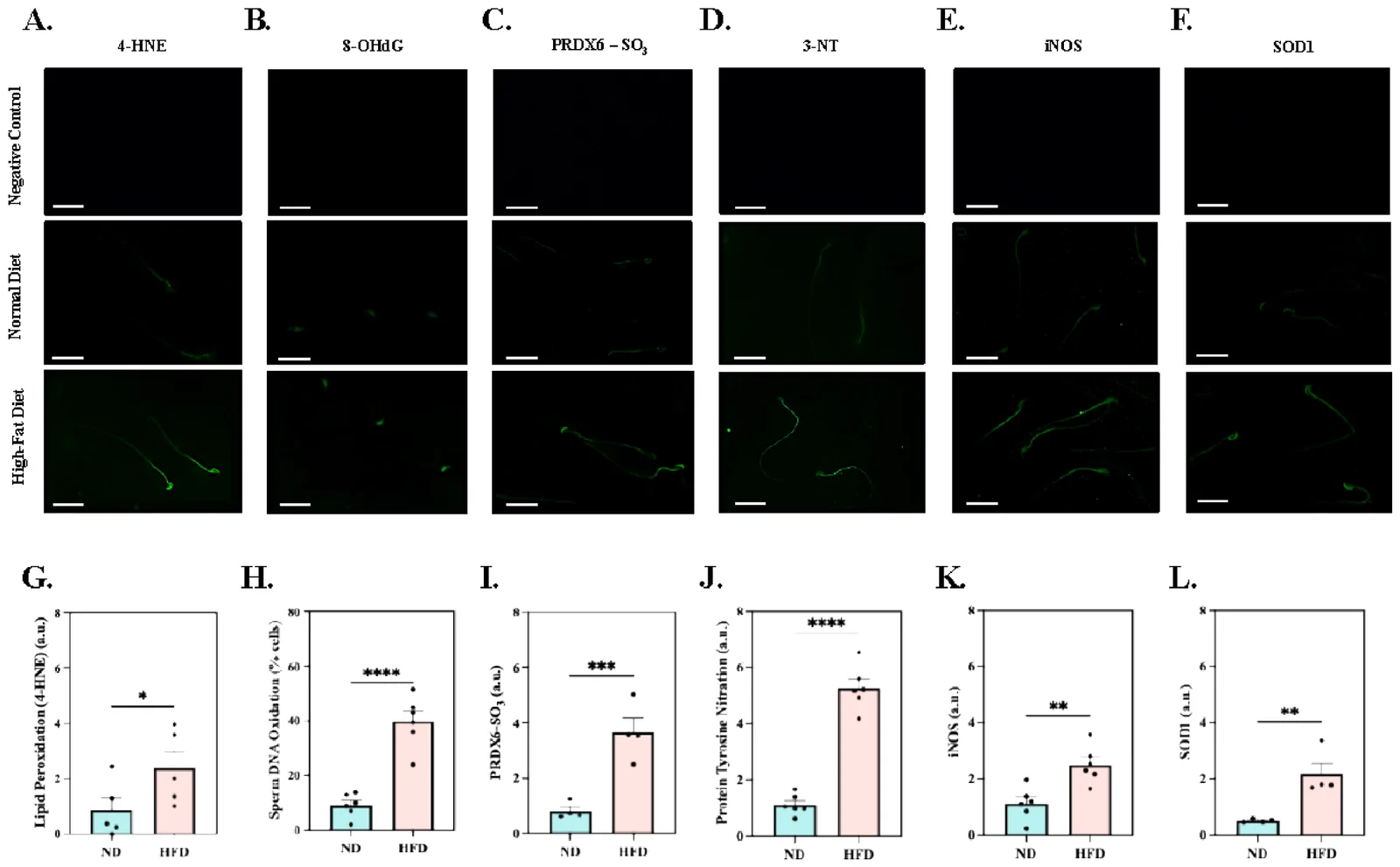
Levels of (**A**) lipid peroxidation (4-hydroxynoneal (4-HNE) labelling), (**B**) DNA oxidation (8-OHdG labelling), (**C**) hyperoxidized PRDX6 (PRDX6-SO3 labelling), (**D**) 3-nitrotyrosine (3-NT labelling), (**E**) inducible nitric oxide synthase (iNOS labelling), and (**F**) superoxide dismutase 1 (SOD1 labelling) in spermatozoa from high-fat diet (HFD)-fed mice. (**G**–**L**). Negative controls were done incubating the samples with secondary antibody alone. Levels of each parameter were quantified by quantifying the average RFI of each parameter in sperm from ND-fed and HFD-fed mice. Protein fluorescence levels are expressed as arbitrary units (a.u.). More than 200 spermatozoa were analyzed per sample. Data are expressed as mean  ±  SEM. Statistical analysis was performed using Student’s *t*-test. (*n* = 4–5 male mice per group). * *p* ≤ 0.05, ** *p* ≤ 0.01, *** *p* ≤ 0.001 and **** *p* ≤ 0.0001. Scale bar = 25 µm.

To further characterize sperm quality, we then determined parameters of oxidative stress in epididymal spermatozoa (Figure 11). Lipid peroxidation was assessed by measuring levels of 4-hydroxynonenal (4-HNE), a byproduct of lipid peroxidation. Immunocytochemical analysis of non-permeabilized spermatozoa revealed a significant 3.4-fold increase in 4-HNE levels in sperm from HFD-fed mice compared to ND-fed controls. Strong labelling was observed in the midpiece and sperm head, particularly within the acrosome (Figure S5), whereas weaker labelling was detected along the flagellum.

Sperm DNA oxidation was assessed by examining levels of 8-hydroxy-2’-deoxyguanosine (8-OHdG) in permeabilized spermatozoa from ND-fed and HFD-fed mice. Immunocytochemical analysis revealed a significantly higher, 4.7-fold increase in the percentage of 8-OHdG-positive sperm in HFD-fed mice compared to ND controls (Figure 11). Strong labelling was located in the sperm head (Figure S5). After observing increased oxidative stress markers in HFD-fed mice, we subsequently examined markers of nitrosative stress. Inducible nitric oxide synthase (iNOS), an enzyme responsible for nitric oxide (NO) production, was assessed by immunocytochemistry of permeabilized sperm. HFD spermatozoa showed significantly higher iNOS levels (2.2-fold) than those from ND-fed mice (Figure 11). Strong labelling was primarily localized to the sperm head and midpiece, with weaker labelling observed along the flagellum (Figure S5).

Because NO can react with superoxide anions to form peroxynitrite, which causes protein tyrosine nitration, we examined 3-nitrotyrosine (3-NT) levels as a marker of protein nitration. Immunolabelling of non-permeabilized sperm revealed strong staining in the midpiece, with lower signal detected in the head and the flagellum (Figure S5). Quantification showed that HFD-fed mice exhibited significant, 5-fold-higher levels of 3-NT than ND controls (Figure 11).

Given the increase in oxidative and nitrosative stress following HFD treatment, we evaluated the status of antioxidant enzymes in spermatozoa. Under excessive oxidative stress, the thiol group at the active site of PRDX6 can become oxidized to a sulfonic group (-SO_3_), rendering the enzyme inactive. Immunocytochemical analysis revealed a significant 4.3-fold increase in levels of hyperoxidized PRDX6 (PRDX6-SO_3_) in permeabilized spermatozoa from HFD-fed mice compared to ND controls (Figure 11). In HFD sperm, strong labelling was observed in the acrosome, with moderate labelling in the midpiece and along the flagellum (Figure S5). In contrast, ND sperm exhibited a distinct staining pattern, with weak labelling in the midpiece and moderate labelling in the principal piece.

Furthermore, permeabilized spermatozoa from HFD-fed mice showed significant, 5-fold-higher SOD1 levels than in ND controls (Figure 11). SOD is an essential antioxidant enzyme in spermatozoa that catalyzes the dismutation of O_2_^•−^ into H_2_O_2_ and oxygen. In both groups, strong SOD1 labelling was observed in the acrosome of the sperm head (Figure S5). However, HFD sperm displayed moderate labelling in the midpiece and weaker labelling along the flagella, whereas ND sperm showed weak labelling throughout the principal piece.

**Figure 12:**
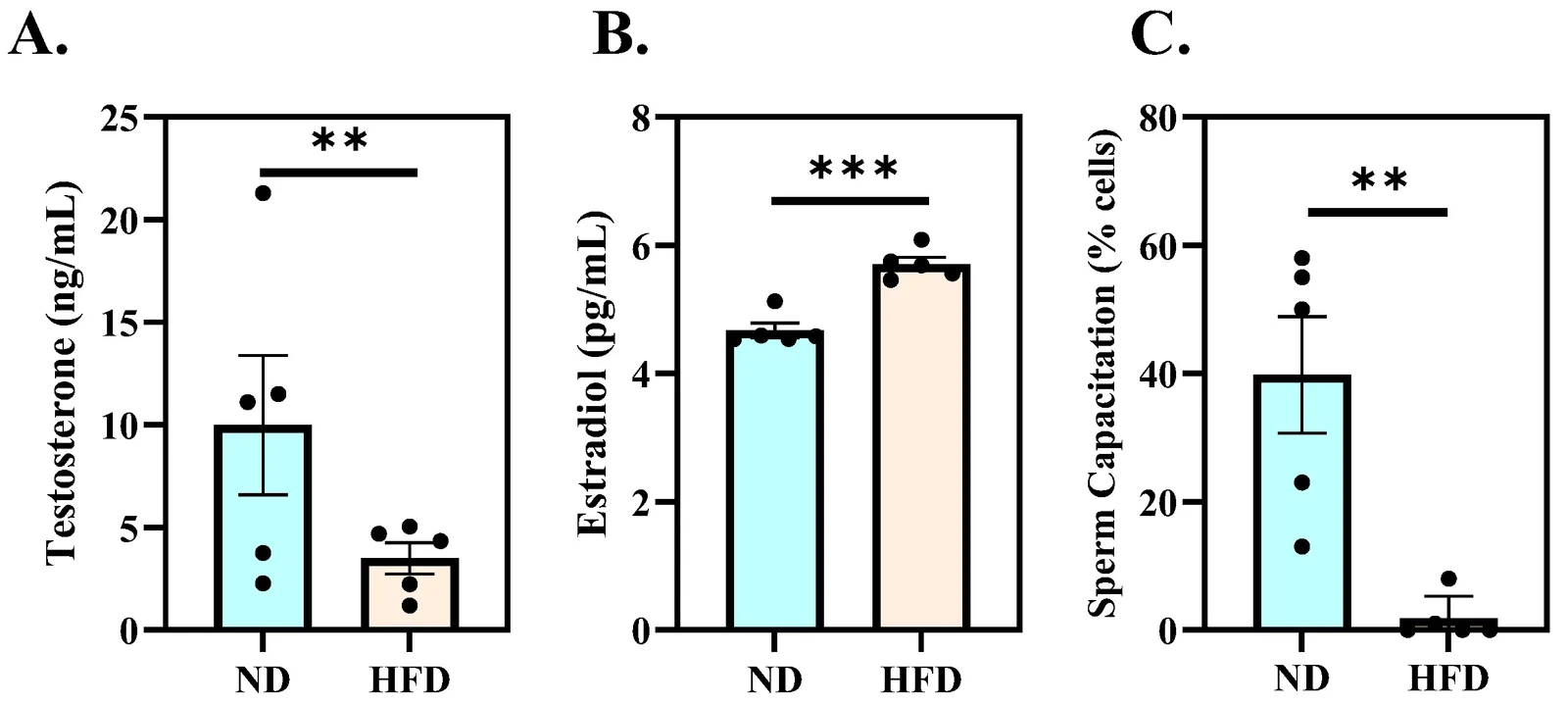
Levels of (**A**) total testosterone and (**B**) estradiol in serum. (**C**) Percentage of sperm capacitation. Levels of hormones were assessed by competitive enzyme-linked immunosorbent assay. Absence of acrosome (Giemsa-negative stain) in spermatozoa corresponds to a reacted acrosome and is a marker of capacitation. More than 200 Giemsa-stained spermatozoa were analyzed per sample using bright-field microscopy. Data are expressed as mean  ±  SEM. Statistical analysis was performed using Student’s *t*-test. (*n* = 4–5 male mice per group). ** *p* ≤ 0.01 and *** *p* ≤ 0.001.

Total testosterone levels were 3.2-fold lower and estradiol levels 1.2-fold higher in HFD-fed males than in ND-fed controls (Figure 12A,B). The percentage of sperm capacitation was 20.7-fold lower in HFD-fed males compared to ND-fed controls (Figure 12C).

## 4. Discussion

In this study, we investigated the impact of gut microbial dysbiosis on male reproductive function. By integrating microbiome profiling with lipidomic and functional sperm analyses, our findings support a gut–testis axis. To our knowledge, we are the first to identify gut microbiome-derived lipids as a potential metabolic link between the gut microbiota and the testis. More specifically, HFD-induced gut microbial dysbiosis promotes alterations in microbial metabolite and sphingolipid production that contribute to systemic oxidative stress and impair sperm function.

Consistent with previous studies, HFD feeding reduced gut microbial alpha diversity, altered community structure, and produced a clear separation between dietary groups in beta-diversity analyses (Figure 1). We also found that HFD-fed mice exhibited an increased Bacillota-to-Bacteroidota ratio and enrichment of several Bacillota families, including *Erysipelatoclostridiaceae, Peptococcaceae, Lachnospiraceae,* and *Oscillospiraceae* (Figure 2).

At the family and genus levels, HFD reduced *Erysipelotrichaceae* and its member *Dubosiella*; *Dubosiella* is a murine intestinal genus belonging to the *Erysipelotrichaceae* family [37,38,39], and reduced *Dubosiella* abundance has also been reported in HFD-fed mice [40]. The concomitant reduction in *Erysipelotrichaceae* and *Dubosiella* in our mice therefore indicates a consistent taxonomic shift within this bacterial lineage. HFD also increased the genus *Akkermansia*; however, *A. muciniphila* was not significantly altered, suggesting that the genus-level change does not reflect a specific enrichment of this species. Together, these taxon-specific changes further illustrate the restructuring of the gut microbial community induced by HFD and confirm previously reported patterns of HFD-induced gut dysbiosis associated with obesity, metabolic dysfunction, inflammation, and impaired intestinal barrier integrity [41,42,43,44] (Figure 2B).

Additionally, HFD-fed mice exhibited a significant depletion of *Muribaculaceae* and *Rikenellaceae* (Figure 2B)*,* two key bacterial families within the *Bacteroidota* phylum that are strongly associated with gut homeostasis and contribute to colonization resistance by limiting the expansion of pathogenic microbes [45]. Reduced abundance of *Muribaculaceae* has been consistently associated with obesity, IBD, and type 2 diabetes, whereas restoration of this family has been linked to improvements in both metabolic and intestinal health [46,47]. Similarly, members of the *Rikenellaceae* family contribute to gut and metabolic health by producing SCFAs, particularly acetate and propionate, which help regulate obesity-related metabolic processes [48]. *Rikenellaceae* also include hydrogen-producing bacteria, a feature that may help protect host tissues from oxidative stress-induced damage [49]. Altogether, these findings suggest that HFD reduces beneficial bacterial families that produce microbiota-derived metabolites involved in maintaining intestinal and metabolic homeostasis.

We observed reduced butyric acid levels in HFD-fed mice, which may also directly affect testicular development and function. Studies using germ-free mice have demonstrated that the absence of gut microbiota-derived butyric acid is associated with impaired testicular development, reduced serum gonadotropin levels (FSH and LH), delayed seminiferous tubule lumen formation, and increased blood–testis barrier (BTB) permeability through reduced expression of tight junction proteins such as Occludin and E-Cadherin [26]. This loss of BTB integrity disrupts the testis’ immune-privileged environment, allowing immune cell infiltration and exacerbating spermatogenic dysfunction [50]. Conversely, restoration of butyric acid production reversed these defects [51]. Accordingly, reduced butyric acid may represent one mechanism linking HFD-induced gut dysbiosis to the impaired sperm quality observed in our study.

Acetic acid was significantly elevated in HFD-fed mice, reflecting the enhanced fermentation activity by *Lachnospiraceae*. Although acetate contributes to normal energy homeostasis [52], elevated acetic acid levels have been reported in obese individuals and animal models of obesity, as they are associated with increased lipogenesis, adiposity, inflammation and metabolic syndrome [53]. HFD-fed mice also exhibited significantly elevated levels of isocaproic acid and hexanoic acid (Figure 3). Isocaproic acid is generated through the bacterial fermentation of branched-chain amino acids [54], suggesting enhanced proteolytic fermentation under HFD conditions. This finding is consistent with the increased abundance of *Peptococcaceae* observed in our study (Figure 2B), as excessive protein fermentation impairs intestinal epithelial integrity and promotes inflammation [55].

We also observed extensive remodelling of the stool, blood, and testis lipidome following HFD treatment (Figure 5, Figure 6, Figure 7 and Figure 8), indicating that gut microbial dysbiosis significantly influenced both intestinal and systemic lipid metabolism. Notably, most of the lipid species enriched in HFD-fed mice were sphingolipids, including ceramides, sphingomyelins, and hexosylceramide, revealing a shift toward a sphingolipid-enriched lipidomic profile. Consistent with previous reports, diseases associated with gut microbial dysbiosis, such as obesity and type 2 diabetes, have been strongly linked to altered sphingolipid metabolism and elevated circulating ceramide levels [56]. In addition, excessive ceramide levels have been shown to promote mitochondrial dysfunction and significantly increase ROS generation via dysregulated electron transport chain [57,58].

Increased ceramides are associated with sperm oxidative damage, including mitochondrial dysfunction and DNA oxidation in human spermatozoa [8]. Obese men exhibit an imbalanced sphingosine-1-phosphate-to-ceramide rheostat and increased levels of pro-apoptotic ceramide in spermatozoa [59]. At the same time, elevated hexosylceramide has been reported in semen lipid profiles from patients with sperm maturation arrest [60]. Therefore, the elevated sphingolipid profile observed in HFD-fed mice may represent an important mechanistic link between gut microbial dysbiosis and the oxidative/nitrosative stress we later identified in HFD-fed mouse spermatozoa (Figure 11 and Figure S5).

Members of the *Bacteroides* genus are capable of de novo sphingolipid synthesis through the enzyme serine palmitoyltransferase (SPT), producing bioactive lipids, including ceramides, sphingomyelins, and hexosylceramides [61]. Consistent with this, several sphingolipid-producing *Bacteroides* species, including *Bacteroides caecimuris* and *Bacteroides massiliensis*, which are strict anaerobic microbes [61,62,63], were enriched in HFD-fed mice and were present at little-to-no abundance in ND-fed mice (Figure 5). Notably, host epithelial cells can take up and metabolize bacterial-derived sphingolipids through endogenous sphingolipid pathways, thereby influencing the enriched host sphingolipid pool [18]. This situation was reflected in our lipidomic datasets, where gut microbiota-associated sphingolipids (SL, SM, Cer, and HexCer), bacterial membrane lipids (NAOrn), and glycerophospholipids (PC and PI) were significantly increased across stool, blood, and testes in our HFD-fed mice. Gut microbiota-associated lipids were identified based on characteristic structural features, including odd-chain fatty acyl groups and iso-branching patterns, which distinguish them from predominantly even-chain host-derived lipids [64,65].

An important finding of this study was the consistent detection of multiple structurally related gut microbiota-associated sphingolipid features across stool, blood, and testis samples (Table 1). Ceramide 17 (a lipid of microbial origin) [18], represented by Cer 17:0;2O/44:7, CerP 17:0;2O/24:1, and Cer 17:0;2O/44:9, was detected in all three biological compartments, supporting the possibility of gut microbiota-associated lipid translocation and/or systemic host remodelling of gut microbial sphingolipids under gut dysbiotic conditions. We detected ceramide-1-phosphate species (CerP 17:0;2O/24:1) in blood, which carries an odd-chain fatty acyl signature consistent with gut bacterial origin, while its phosphorylation reflects host ceramide kinase activity [66], suggesting a combined microbial–host origin. These modified sphingolipids may then be transported systemically via lipoprotein-associated pathways, enabling distribution to peripheral tissues including the testes, as observed in our study (Table 1).

In parallel, we observed reduced levels of several sphingolipid species in stool and blood of HFD-fed mice (Figure 6, Figure 7 and Figure 8). Sphingolipids play essential roles in maintaining sperm membrane fluidity and regulating key signalling events required for capacitation, the acrosome reaction, and sperm–oocyte interaction [8,67]. Therefore, reduced levels of specific sphingolipid species in the testes may promote improper loading of these lipids into the forming spermatozoon, with negative consequences for critical signalling pathways required for normal sperm function and potentially contributing to reproductive dysfunction.

HFD-fed mice exhibited reduced sperm motility, velocity, and kinematic parameters (Figure 9), indicating marked functional impairment and potentially reduced fertilization capacity. These findings are consistent with previous reports linking diet-induced gut microbial dysbiosis to impaired sperm quality in animal models [28,68]. Systemic inflammation, oxidative stress, and mitochondrial dysfunction may contribute to this phenotype, as epididymal spermatozoa from HFD-fed mice displayed significantly increased markers of oxidative and nitrosative stress. Although we did not directly assess mitochondrial function, these results align with our previous finding that exposure to high ceramide concentrations promotes sperm mitochondrial dysfunction, DNA damage, and lipid peroxidation [8]. Further studies are needed to determine whether altered ceramide metabolism and mitochondrial dysfunction directly contribute to the sperm defects induced by HFD feeding.

We further observed a significant increase in spontaneous acrosome reaction (sAR) in HFD-fed mice (Figure 10), indicating impaired acrosomal membrane integrity. sAR occurs prematurely in the absence of a physiological inducer of acrosome reaction, resulting in premature release of hydrolytic enzymes required for zona pellucida penetration and rendering sperm fertilization incompetent [69]. Increased sAR was observed in obese men and HFD-fed mouse models [70,71]. Obesity has been associated with decreased testosterone, altered estradiol levels and increased sperm cholesterol content, all of which may impair sperm membrane stability and contribute to increased sAR levels [70].

To determine whether the increased sAR observed in HFD spermatozoa could be due to alterations in acrosome biogenesis during spermatogenesis, we performed histological evaluation of testis sections stained with Periodic acid–Schiff (PAS). All spermatogenesis stages were present in HFD-fed mice (Figure S4). Furthermore, quantification of acrosome formation in stage VII seminiferous tubules showed no significant differences between groups, indicating that HFD-induced gut microbial dysbiosis did not affect acrosome formation (Figure 10B). Thus, the increased sAR likely did not originate during testicular sperm development but instead arose post-testicularly during epididymal sperm maturation.

Changes in the epididymal environment occur due to HFD exposure [72]. Indeed, the significant increase in cytoplasmic droplet retention (CDR) observed in HFD-fed mice indicates impaired epididymal sperm maturation (Figure 9). During normal epididymal transit, spermatozoa undergo post-testicular maturation involving membrane remodelling, acquisition of motility and, most importantly, the removal of excess residual cytoplasm [73]. Failure to remove cytoplasmic droplets is associated with reduced sperm motility, abnormal head and midpiece morphology, shorter axonemal length, and infertility in men with varicocele [74,75,76]. Spermatozoa with CDR are also highly susceptible to oxidative stress, as studies have shown a positive association with increased lipid peroxidation and disrupted sperm membrane remodelling [77,78].

In parallel, HFD-fed mice exhibited significantly increased levels of the cytosolic antioxidant superoxide dismutase 1 (SOD1) (Figure 11). This finding likely reflects the increased retention of residual cytoplasm (Figure 9), which contains higher amounts of the enzyme than normal spermatozoa. Although SOD1 scavenges O_2_^•−^, its enzymatic activity generates H_2_O_2_, which is highly toxic to spermatozoa [79]. Therefore, increased SOD1 levels do not confer enhanced antioxidant protection. Furthermore, downstream antioxidant systems such as peroxiredoxins (PRDXs), the primary antioxidant defence system in sperm, are required to neutralize increased H_2_O_2_ generation and other ROS such as peroxynitrite (ONOO^−^) [80,81]. Interestingly, we detected increased hyperoxidized PRDX6 (PRDX6-SO_3_) in HFD-fed mouse spermatozoa (Figure 11). Hyperoxidation of PRDX6 occurs under conditions of excessive oxidative stress, resulting in enzymatic inactivation and loss of antioxidant capacity. These findings suggest that antioxidant defences in HFD-fed mouse spermatozoa are not only overwhelmed but also functionally impaired under HFD-induced gut dysbiosis. Increased PRDX6 hyperoxidation has previously been reported in spermatozoa following oxidative stress exposure [82]. The lack of PRDX6 peroxidase activity (knock-in mouse model) and its pharmacological inhibition have been associated with increased mitochondrial ROS production, lipid peroxidation, and DNA damage in spermatozoa [36].

Moreover, we observed sperm DNA damage in our HFD-fed mice, indicated by increased levels of 8-hydroxy-2′-deoxyguanosine (8-OHdG) within the sperm head (Figure 11). Sperm DNA integrity is essential for normal embryo development and offspring health [83]. Therefore, oxidative damage to sperm DNA has been strongly associated with impaired embryo development and increased risk of genetic abnormalities [84]. Hyperoxidation of PRDX6 may also contribute to this phenotype, as loss of PRDX6 activity has been shown to increase oxidative DNA damage in spermatozoa [85].

HFD-fed mice also exhibited significantly increased levels of inducible nitric oxide synthase (iNOS) and protein tyrosine nitration, indicating nitrosative stress (Figure 11 and Figure S4). Although regulated levels of NO are required for normal sperm function, excessive NO production has been associated with impaired sperm motility, inhibition of cellular respiration, mitochondrial dysfunction, and DNA damage, resulting in reduced male fertility [36,85,86,87]. Importantly, O_2_^•−^ generated during oxidative stress can react rapidly with NO to produce ONOO^−^, a highly reactive molecule capable of inducing lipid peroxidation, protein oxidation and protein tyrosine nitration [36,85,88]. Thus, the significantly increased levels of 3-nitrotyrosine (3-NT) observed in HFD spermatozoa (Figure 11) strongly suggest enhanced ONOO^−^ formation and nitrosative protein damage. The elevated nitrosative stress observed in our study may also be exacerbated by impaired PRDX6 activity, as we observed previously in mouse spermatozoa lacking PRDX6 peroxidase activity [36]. In addition, potential targets of tyrosine nitration such as tubulin and glycolytic enzymes found in the fibrous sheath of the sperm flagellum [6] can further contribute to sperm impairments identified in HFD-fed mice.

Oxidative and nitrosative stress impair male fertility [5,36]. The ability of spermatozoa to undergo capacitation, to acquire fertilizing ability, is negatively affected by high levels of sphingolipids like ceramide and sphingosine [8]. The low percentage of capacitation observed in HFD-fed males compared to ND-fed controls indicates that their spermatozoa, carrying significant oxidative damage as shown in Figure 11, fail to acquire fertilizing ability. Altogether, these findings indicate that exposure to high levels of sphingolipids seen in HFD-fed males can impair male fertility. Further studies are necessary to determine whether sphingolipids produced by the gut microbiota dysbiosis can promote male infertility.

Overall, our findings support a mechanistic framework whereby HFD-induced gut microbial dysbiosis contributes to male infertility through alterations in microbial metabolism that promote oxidative stress. Several limitations should be acknowledged. Although our findings support the existence of a gut–testis–epididymis axis, we did not establish causality. This is a limitation of this study, and supplementation with identified microbial-derived metabolites will be necessary to support the existence of such an axis. In addition, we did not directly measure intestinal permeability, systemic endotoxin levels, inflammatory cytokines, or mitochondrial function, limiting our ability to confirm the proposed mechanisms linking gut dysbiosis to oxidative stress and sperm dysfunction. The HFD-mouse model may also limit direct translation to human male infertility, as species-specific differences exist in microbiome composition and reproductive physiology. Furthermore, several bacterial taxa identified in the microbiome analyses remained unclassified, reflecting current limitations in microbial reference databases and potentially overlooking additional functionally relevant microbes. Finally, while the detection of structurally related odd-chain sphingolipids across stool, blood, and testis is consistent with microbial lipid translocation and/or host remodelling, further investigation is needed to confirm these complex interactions between the gut microbiota and the host that may modify sperm quality.

## 5. Conclusions

This study is the first to identify gut microbiome-derived lipids as a potential metabolic link within the gut–testis axis, providing novel insight into mechanisms that may contribute to idiopathic male infertility. Collectively, our findings support a gut–testis axis in which HFD-induced gut microbial dysbiosis alters the microbial lipid profile, contributing to oxidative and nitrosative stress that impairs sperm quality. Moreover, detecting gut microbiota-associated lipid signatures in the testes further supports the role of microbial-derived metabolites in male reproductive dysfunction. Future studies should validate these lipidomic findings using targeted lipidomics and identify the specific microbial-derived metabolites responsible for the observed reproductive phenotype. Supplementation studies with identified microbial-derived lipids are needed to demonstrate that they could impair male fertility.

## Figures and Tables

**Table 1:** Structurally related lipid species annotated across stool, serum, and testis samples ^1^.

Lipid Class	Lipid Species	Sample Origin	Fold Change (HFD vs. ND)	*p*-Value	Shared Across Samples
Ceramide	Cer 17:0;2O/44:7	Stool	0.69283	0.038026	Stool, Serum, Testis
Ceramide Phosphate	CerP 17:0;2O/24:1	Serum	0.64761	0.0082951	Stool, Serum, Testis
Ceramide	Cer 17:0;2O/44:9	Testis	1.1415	0.0009	Stool, Serum, Testis
Hexosylceramide	Hex2Cer 16:2;2O/17:2	Stool	−3.9419	0.0087247	Stool, Serum
Hexosylceramide	Hex2Cer 16:2;2O/14:1	Serum	2.9083	3.8337 × 10^−8^	Stool, Serum
Hexosylceramide	HexCer 8:0;2O/44:6	Stool	−2.6108	0.0000151	Stool, Testis
Hexosylceramide	HexCer 8:0;2O/42:6	Testis	1.1172	0.0029	Stool, Testis
Sphingomyelin	SM 9:0;2O/42:10	Stool	1.5934	0.00031715	Stool, Serum
Sphingomyelin	SM 9:0;2O/38:10	Serum	1.0874	0.00066834	Stool, Serum
Hydroxy-Ceramide	SL 11:1;O/18:2;O	Stool	3.7427	0.0014448	Stool, Serum
Hydroxy-Ceramide	SL 11:1;O/16:2;O	Serum	0.87393	0.000016629	Stool, Serum
Phosphatidylcholine	PC O-41:1	Stool	1.1133	0.0000781	Stool, Serum
Phosphatidylcholine	PC O-41:6	Serum	1.069	0.014957	Stool, Serum
Phosphatidylinositol	PI 41:0	Stool	−3.6622	0.0026503	Stool, Serum
Phosphatidylinositol	PI 41:0	Serum	1.2724	0.012947	Stool, Serum

^1^ Lipid species putatively annotated by UHPLC-MS/MS analysis of stool, serum, and testicular tissue from 8-week-old HFD-fed and ND-fed mice. Lipids listed share similar backbone structures but differ in fatty acyl chain composition.

## Data Availability

The raw data supporting the conclusions of this article will be made available by the authors upon request.

## Supplementary Materials

The following supporting information can be downloaded at https://www.mdpi.com/article/10.3390/antiox15101281/s1, Figure S1: Body and organs weights; Figure S2: Relative abundance (A) Phylum and (B) Genus levels. Figure S3: Correlation heat maps of gut microbiome taxa and lipidomic features in high-fat diet (HFD)-fed and normal diet (ND)-fed mice; Figure S4: High-fat diet (HFD) does not affect stages of spermatogenesis; Figure S5: Representative pictures of localization and levels of lipid peroxidation (4-hydroxynoneal (4-HNE labelling), DNA oxidation (8-OHdG labelling), hyperoxidized PRDX6 (PRDX6-SO3 labelling), 3-nitrotyrosine (3-NT labelling), inducible nitric oxide synthase (iNOS labelling), and superoxide dismutase 1 (SOD1 labelling) in spermatozoa from high-fat diet (HFD)-fed mice. Figure S6. Representative lipid structures from Table 1. Table S1: Food intake (g/day/mice). Table S2: Quantification of testicular cells in seminiferous tubles at Stage VII.
