# Supplementary material for: Lipidomic Remodelling and Low Sperm Quality Associated with High-Fat Diet-Induced Gut Microbial Dysbiosis in Mice
Source: Antioxidants (Basel). 2026 Oct 3;15(10):1281. doi: 10.3390/antiox15101281 (PMC13700696; doi:10.3390/antiox15101281)
Supplement: Supplementary file 1 [file antioxidants-15-01281-s001.zip › antioxidants-4554616-supplementary.pdf]

Table S1. Food intake (g/day/mice)

Group	Week 4	Week 5	Week 6	Week 7	Week 8
ND	1.76±0.05	2.20±0.03	2.36.4±0.04	2.10±0.06	2.32±.12
HFD	1.82±0.04	2.04±0.05	2.20±0.06	2.06±0.05	2.36±0.13

No differences between the groups. Two-Way ANOVA, n = 39 (ND) and 38 (HFD) male mice; $p>0.05$.

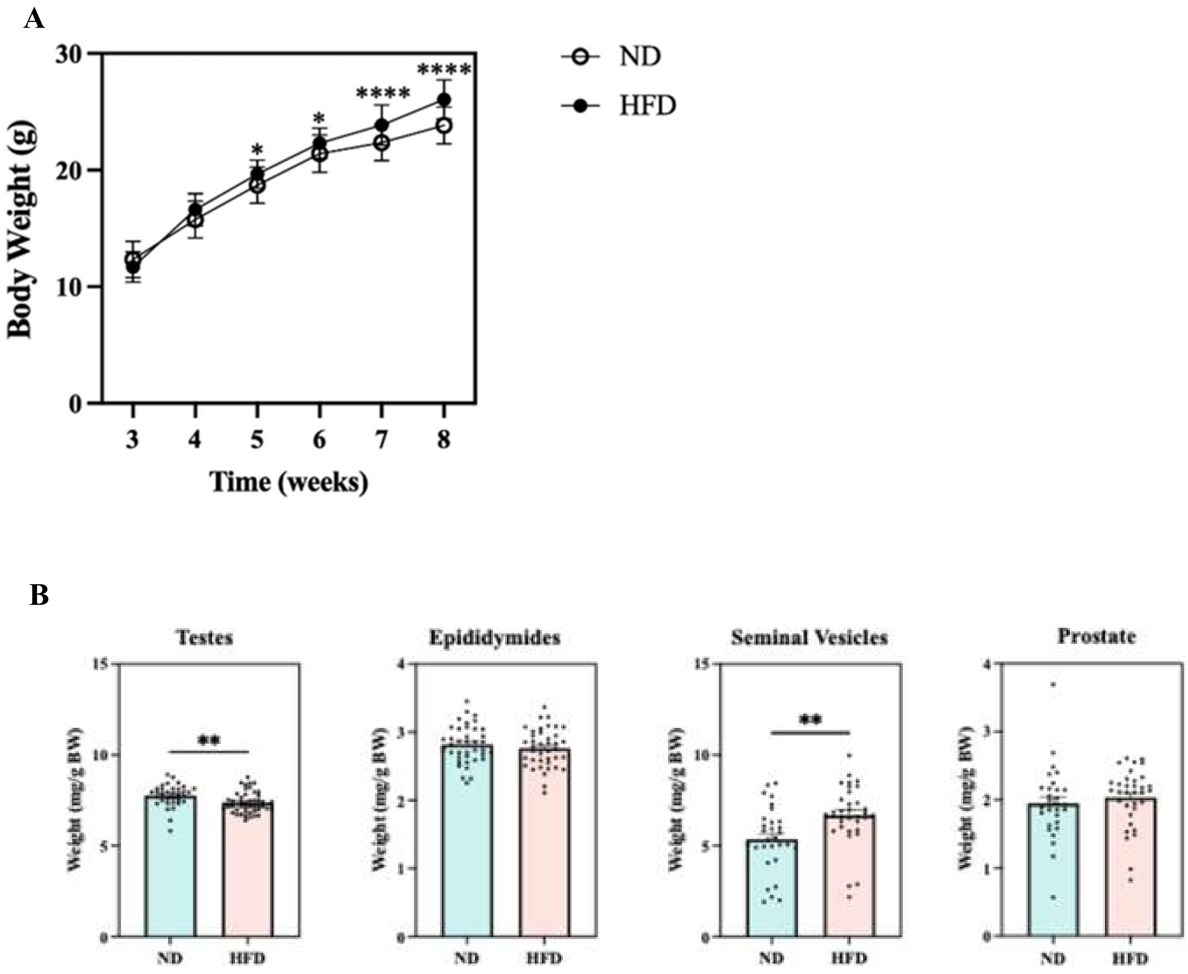

Figure S1. Body and organ weights.

(A) Body weight progression of normal diet (ND)-fed and high-fat diet (HFD)-fed mice. Mice were weighed weekly from 3 to 8 weeks of age. (B) Organ weights of 8-week-old male mice fed a normal diet (ND) or high-fat diet (HFD). Weights of testes, epididymides, seminal vesicles (with fluid), and prostate in 8-week-old HFD- and ND-fed mice. Data are presented as mean ± SEM. Statistical analysis was performed using Student's *t*-test, $n = 39$ (ND) and 38 (HFD) male mice. *P≤0.05, **P≤0.001, ***P≤0.001.

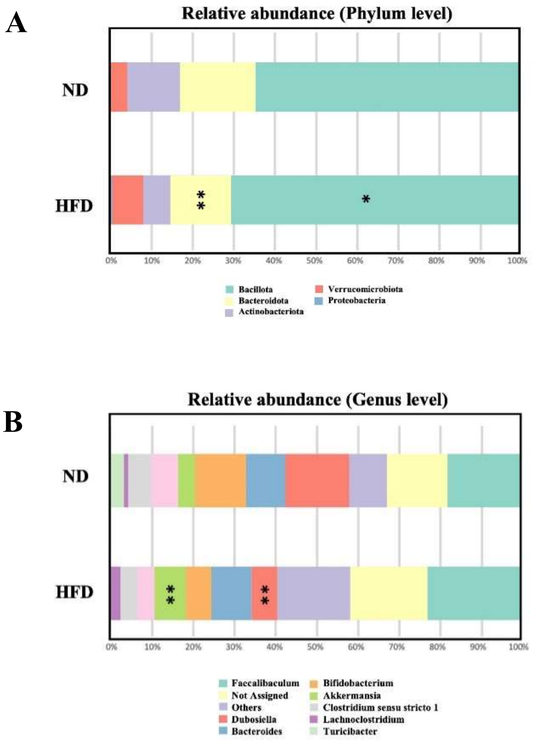

Figure S2. Relative abundance (A) Phylum and (B) Genus levels. Statistical analysis was performed using Student's t-test. Data are expressed as mean ± SEM. The relative abundance of bacterial families. Statistical analysis was performed using the Mann-Whitney U test. n = 10 (ND) and 9 (HFD) male mice. *P≤0.05, **P≤0.01.

A

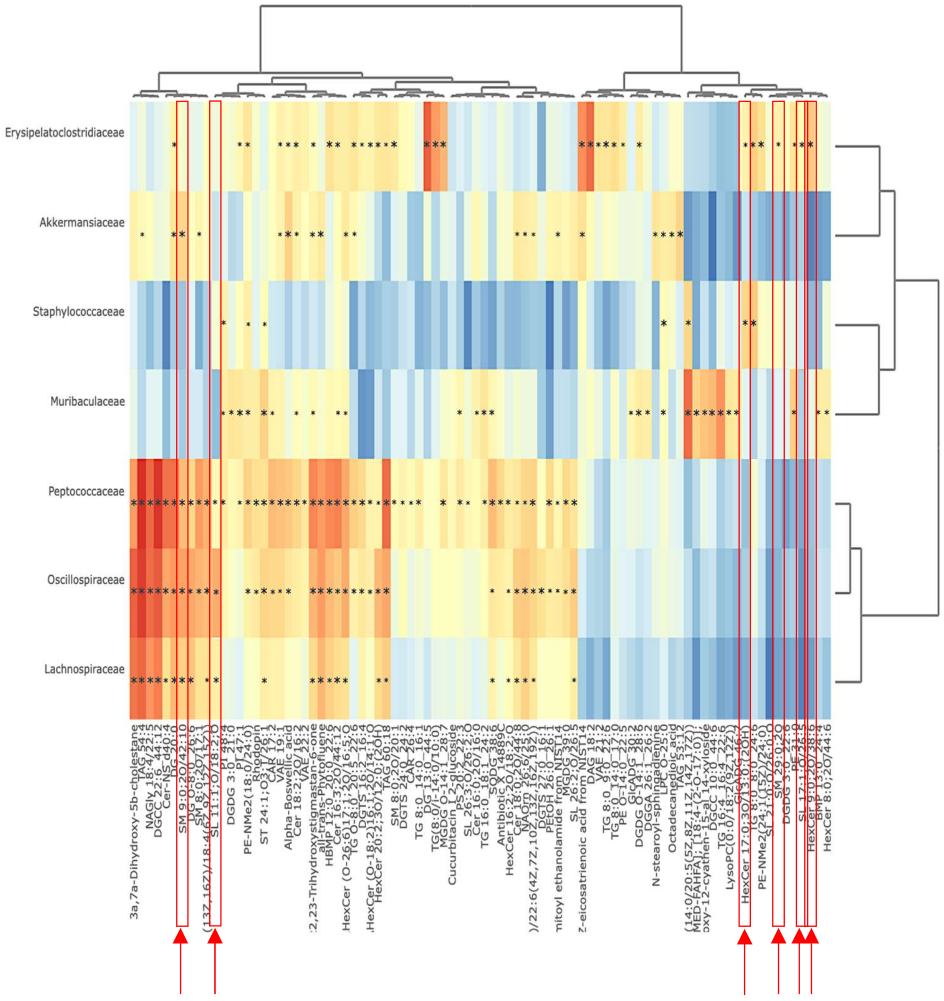
Erysipelatoclostridiaceae
Akkermansiaceae
Staphylococcaceae
Muribaculaceae
Peptococcaceae
Oscillospiraceae
Lachnospiraceae

B

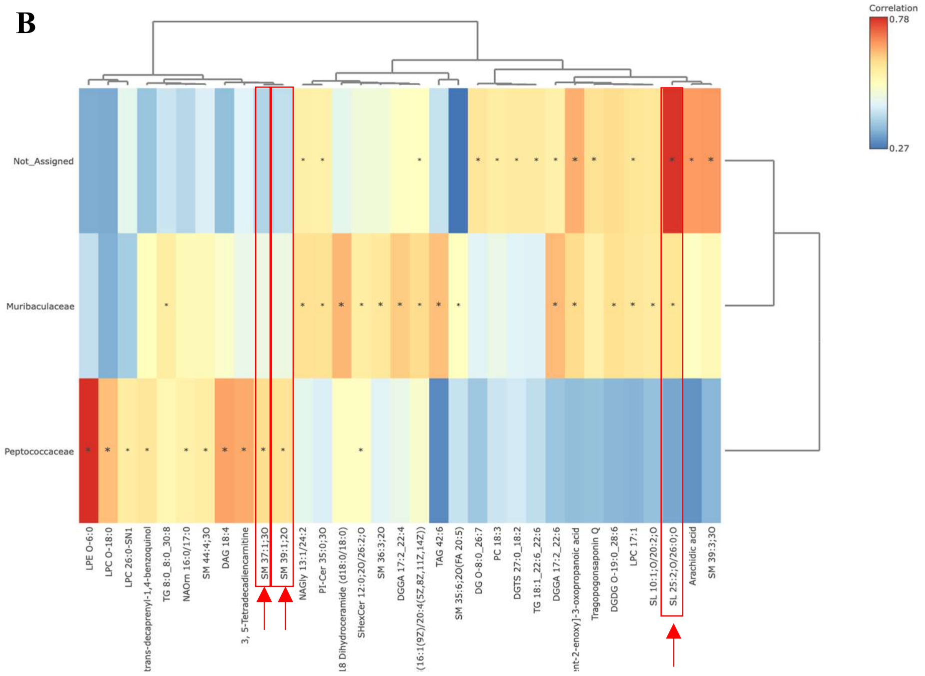

C

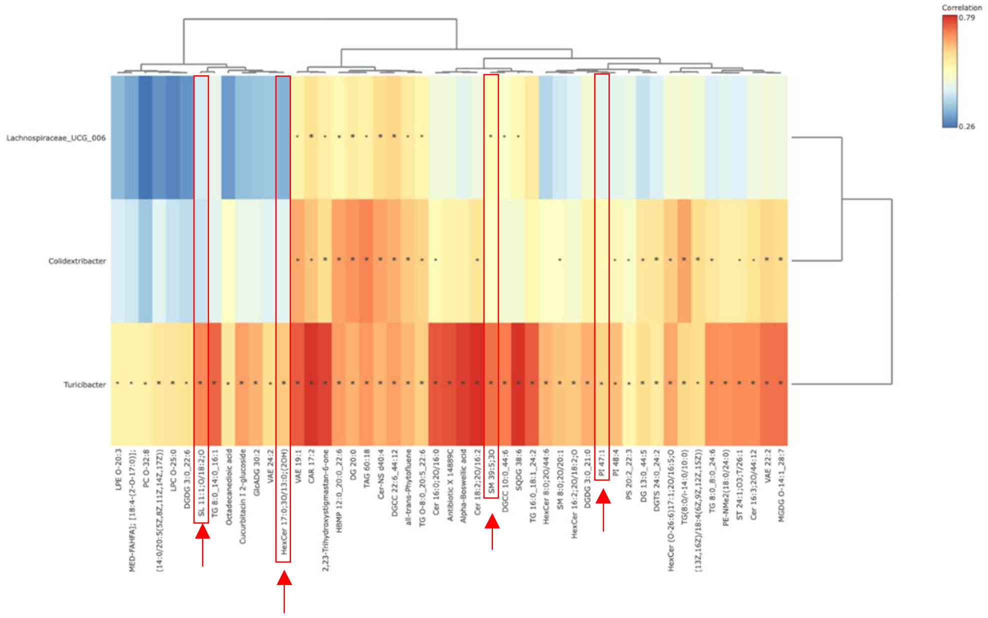

Figure S3. Correlation heat maps of gut bacterial families and lipidomic features in normal diet (ND)- and high-fat diet (HFD)-fed mice. The relative abundance of bacterial families, determined by 16S rRNA gene sequencing of stool samples and taxonomic classification using the SILVA database, was correlated with annotated lipid features detected by UPLC–MS/MS in (A) stool and (B) serum samples from 8-week-old ND- and HFD-fed mice. (C) Correlations in stools at genus level. Correlations were calculated across the combined ND and HFD cohorts using Spearman correlation analysis. In panel A, selected putatively microbiota-associated lipids are highlighted, including SM 9:0;2O/42:1;O, SL 11:1;O/18:2;O, HexCer 17:0;3O/13:0;(2OH), HexCer 9:0;2O/38:6, SM 29:0;2O, and SL 17:1;O/26:5. In panel B, the serum lipid features SM 37:1;3O and SM 39:1;2O, and in panel C SL 11:1;O/18:2;O, HexCer 17:0;3O/13:0;(2OH), SM 39:5;3O, and PI 47:1 are highlighted. Microbiome data were [TSS-normalized and subsequently CLR-transformed], and lipidomic data were log_{10}-transformed before correlation analysis. Rows represent bacterial families, and columns represent annotated lipid features. The colour scale represents the correlation coefficient, with blue and red indicating negative and positive correlations, respectively. Asterisks indicate statistically significant correlations based on nominal $p < 0.05$.

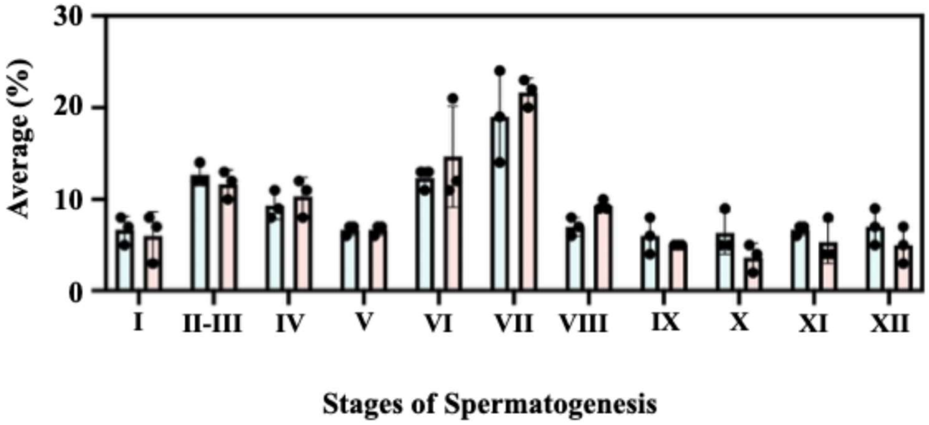

Figure S4. High-fat diet (HFD) does not affect stages of spermatogenesis. A. Quantification of all stages of spermatogenesis in PAS-stained seminiferous tubules (stages I-XII) in ND (light blue bars) and HFD (pink bars) mice. More than 200 tubules were scored for each group. Data are expressed as mean ± SEM. Statistical analysis was performed using Student's *t*-test. The p-value threshold was $p \leq 0.05$. ($n = 3$ mice per group).

Table S2. Quantification of testicular cells in seminiferous tubles at Stage VII

Group	Spermatogonia +Preleptotene spermatocytes	Round spermatids	Sertoli Cells
ND	42.11±3.40	141.67±3.95	17.89±0.48
HFD	41.44±2.17	148.11±4.07	16.11±0.75

Data are expressed as mean ± SEM. Statistical analysis was performed using Student's *t*-test. (n = 3 mice per group).

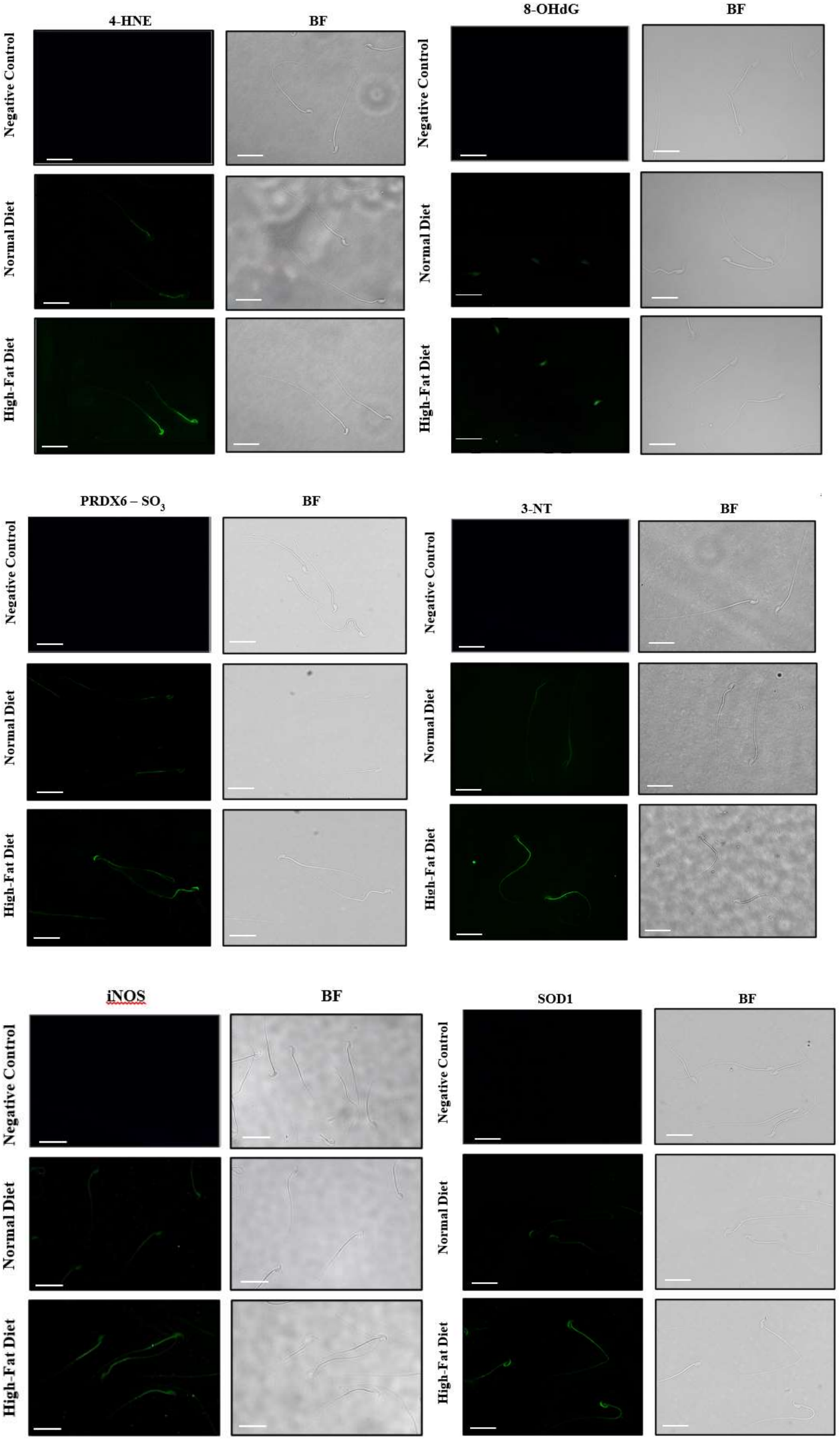

4-HNE
BF
8-OHdG
BF
Negative Control
Normal Diet
High-Fat Diet
Negative Control
Normal Diet
High-Fat Diet
PRDX6 – SO_3
BF
3-NT
BF
Negative Control
Normal Diet
High-Fat Diet
Negative Control
Normal Diet
High-Fat Diet
iNOS
BF
SOD1
BF
Negative Control
Normal Diet
High-Fat Diet
Negative Control
Normal Diet
High-Fat Diet

Figure S5. Representative pictures of localization and levels of lipid peroxidation (4-hydroxynoneal (4-HNE labeling), DNA oxidation (8-OHdG labeling), hyperoxidized PRDX6 (PRDX6-SO_3 labeling) 3-nitrotyrosine (3-NT labeling), inducible nitric oxide synthase (iNOS labeling), and superoxide dismutase 1 (SOD1 labeling) in spermatozoa from high-fat diet (HFD)-fed mice. BF: brightfield images of ND and HFD sperm showing 4-HNE labelling. **(B)** Quantification of the average RFI of 4-HNE in sperm from ND-fed and HFD-fed mice. Protein fluorescence levels are expressed as arbitrary units (a.u.). >200 spermatozoa were analyzed per sample. Data are expressed as mean ± SEM. Statistical analysis was performed using Student's *t*-test. *P≤0.05. P-value threshold was p<0.05. Scale bar = 25μm. (*n* = 5 per group).

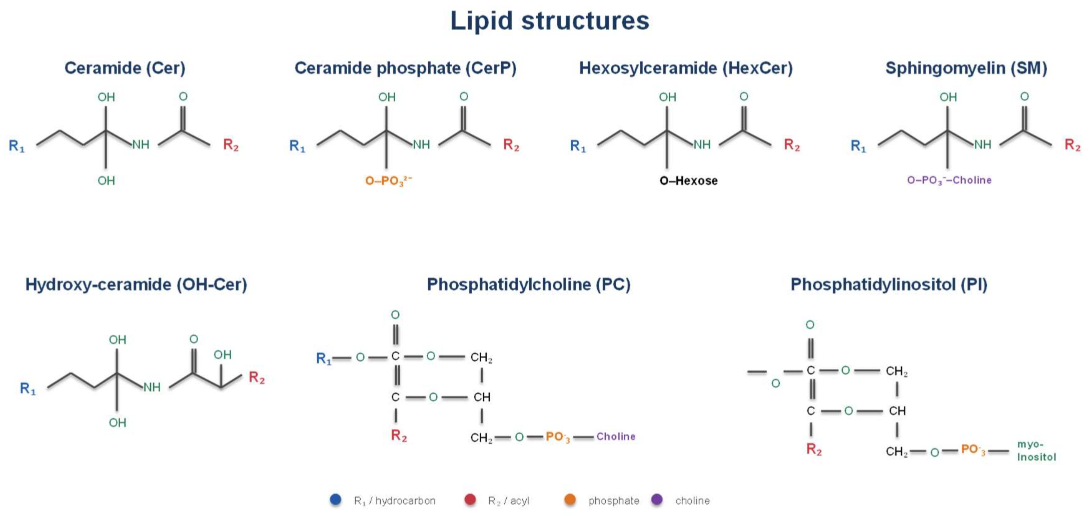

Figure S6. Representative lipid structures from table 1
